# Supplementary material for: Synthesis and Biological Evaluation of 1-(2-(6-Methoxynaphthalen-2-yl)-6-methylnicotinoyl)-4-Substituted Semicarbazides/Thiosemicarbazides as Anti-Tumor Nur77 Modulators
Source: Molecules. 2022 Mar 4;27(5):1698. doi: 10.3390/molecules27051698 (PMC8911927; doi:10.3390/molecules27051698)
Supplement: Supplementary file 1 [file molecules-27-01698-s001.zip › molecules-1603821-supplementary.pdf]

molecules

MDPI

Supplementary Materials

Synthesis and Biological Evaluation of 1-(2-(6-Methoxynaphthalen-2-yl)-6-Methylnicotinoyl)-4-Substituted Semicarbazides/Thiosemicarbazides as Anti-Tumor Nur77 Modulators

Hongyu Hu [1,2,3,4,5,†], Jiangang Huang [3,†], Yin Cao [3,†], Zhaolin Zhang [4], Fengming He [3], Xianfu Lin [1], Qi Wu [1,*] and Shengxian Zhao [2,5*]

[1] Department of Chemistry, Zhejiang University, Hangzhou 310027, China; huhongyu22@126.com (H.H.); xflin@zju.edu.cn (X.L.)
[2] College of Science and Technology, Ningbo University, Cixi 315302, China; zhaoshengxian@nbu.edu.cn
[3] School of Pharmaceutical Sciences, Xiamen University, South Xiang-An Road, Xiamen 361102, China; jxsdhjg@163.com (J.H.); 32320171153273@stu.xmu.edu.cn (Y.C.); fengminghe@stu.xmu.edu.cn (F.H.)
[4] Xingzhi College, Zhejiang Normal University, Lanxi 321004, China; ala_1208@163.com
[5] Zhejiang Apeloa Kangyu Pharmaceutical Co., Ltd., Dongyang 322118, China
* Correspondence: wuqi1000@163.com (Q.W.); zhaoshengxian@nbu.edu.cn (S.Z.); Tel.: +86-0571-8795-3001 (Q.W.)
† These authors contributed equally to this work.

Content

Figure S1. ^{1}H-NMR Spectrum of compound **9a** 4

Figure S2. ^{13}C-NMR Spectrum of compound **9a** 4

Figure S3. ESI-HRMS of compound **9a** 5

Figure S4. ^{1}H-NMR Spectrum of compound **9b** 5

Figure S5. ^{13}C-NMR Spectrum of compound **9b** 6

Figure S6. ESI-HRMS of compound **9b** 6

Figure S7. ^{1}H-NMR Spectrum of compound **9c** 7

Figure S8. ^{13}C-NMR Spectrum of compound **9c** 7

Figure S9. ESI-HRMS of compound **9c** 8

Figure S10. ^{1}H-NMR Spectrum of compound **9d** 8

Figure S11. ^{13}C-NMR Spectrum of compound **9d** 9

Figure S12. ESI-HRMS of compound **9d** 9

Figure S13. ^{1}H-NMR Spectrum of compound **9e** 10

Figure S14. ^{13}C-NMR Spectrum of compound **9e** 10

Figure S15. ESI-HRMS of compound **9e** 11

Figure S16. ^{1}H-NMR Spectrum of compound **9f** 11

Figure S17. ^{13}C-NMR Spectrum of compound **9f** 12

Figure S18. ESI-HRMS of compound **9f** 12

Figure S19. ^{1}H-NMR Spectrum of compound **9g**......13
Figure S20. ^{13}C-NMR Spectrum of compound **9g**......13
Figure S21. ESI-HRMS of compound **9g**......14
Figure S22. ^{1}H-NMR Spectrum of compound **9h**......14
Figure S23. ^{13}C-NMR Spectrum of compound **9h**......15
Figure S24. ESI-HRMS of compound **9h**......15
Figure S25. ^{1}H-NMR Spectrum of compound **9i**......16
Figure S26. ^{13}C-NMR Spectrum of compound **9i**......16
Figure S27. ESI-HRMS of compound **9i**......17
Figure S28. ^{1}H-NMR Spectrum of compound **9j**......17
Figure S29. ^{13}C-NMR Spectrum of compound **9j**......18
Figure S30. ESI-HRMS of compound **9j**......18
Figure S31. ^{1}H-NMR Spectrum of compound **9k**......19
Figure S32. ^{13}C-NMR Spectrum of compound **9k**......19
Figure S33. ESI-HRMS of compound **9k**......20
Figure S34. ^{1}H-NMR Spectrum of compound **9l**......20
Figure S35. ^{13}C-NMR Spectrum of compound **9l**......21
Figure S36. ESI-HRMS of compound **9l**......21
Figure S37. ^{1}H-NMR Spectrum of compound **9m**......22
Figure S38. ^{13}C-NMR Spectrum of compound **9m**......22
Figure S39. ESI-HRMS of compound **9m**......23
Figure S40. ^{1}H-NMR Spectrum of compound **9n**......23
Figure S41. ^{13}C-NMR Spectrum of compound **9n**......24
Figure S42. ESI-HRMS of compound **9n**......24
Figure S43. ^{1}H-NMR Spectrum of compound **9o**......25
Figure S44. ^{13}C-NMR Spectrum of compound **9o**......25
Figure S45. ESI-HRMS of compound **9o**......26
Figure S46. ^{1}H-NMR Spectrum of compound **9p**......26
Figure S47. ^{13}C-NMR Spectrum of compound **9p**......27
Figure S48. ESI-HRMS of compound **9p**......27
Figure S49. ^{1}H-NMR Spectrum of compound **9q**......28
Figure S50. ^{13}C-NMR Spectrum of compound **9q**......28
Figure S51. ESI-HRMS of compound **9q**......29

Figure S52. ^{1}H-NMR Spectrum of compound **9r**............29
Figure S53. ^{13}C-NMR Spectrum of compound **9r**............30
Figure S54. ESI-HRMS of compound **9r**............30
Figure S55. ^{1}H-NMR Spectrum of compound **9s**............31
Figure S56. ^{13}C-NMR Spectrum of compound **9s**............31
Figure S57. ESI-HRMS of compound **9s**............32
Figure S58. ^{1}H-NMR Spectrum of compound **9t**............32
Figure S59. ^{13}C-NMR Spectrum of compound **9t**............33
Figure S60. ESI-HRMS of compound **9t**............33
Figure S61. ^{1}H-NMR Spectrum of compound **9u**............34
Figure S62. ^{13}C-NMR Spectrum of compound **9u**............34
Figure S63. ESI-HRMS of compound **9u**............35
Figure S64. ^{1}H-NMR Spectrum of compound **9v**............35
Figure S65. ^{13}C-NMR Spectrum of compound **9v**............36
Figure S66. ESI-HRMS of compound **9v**............36
Figure S67. ^{1}H-NMR Spectrum of compound **9w**............37
Figure S68. ^{13}C-NMR Spectrum of compound **9w**............37
Figure S69. ESI-HRMS of compound **9w**............38

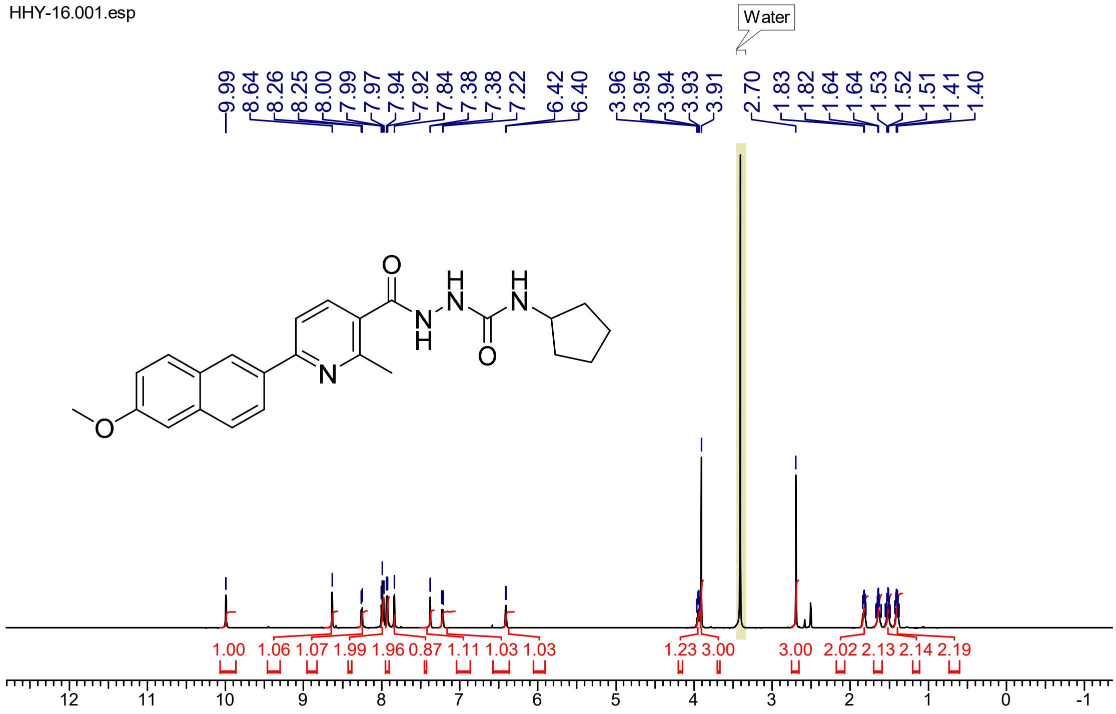

Figure S1. ^{1}H-NMR Spectrum of compound **9a.**

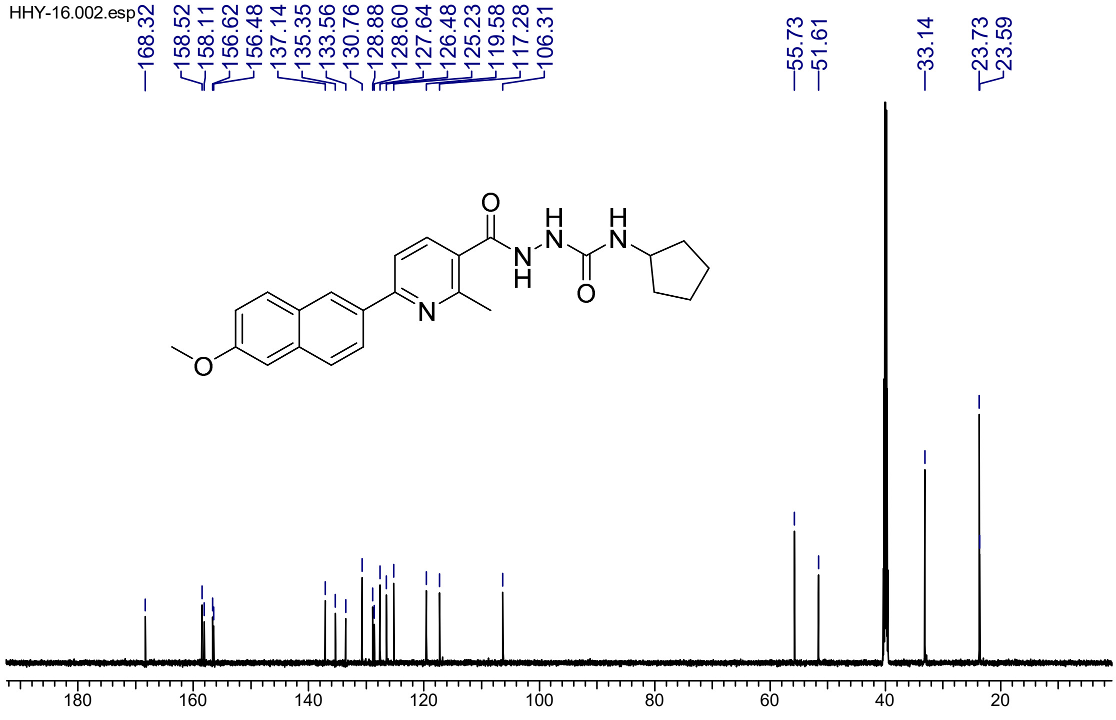

Figure S2. ^{13}C-NMR Spectrum of compound **9a.**

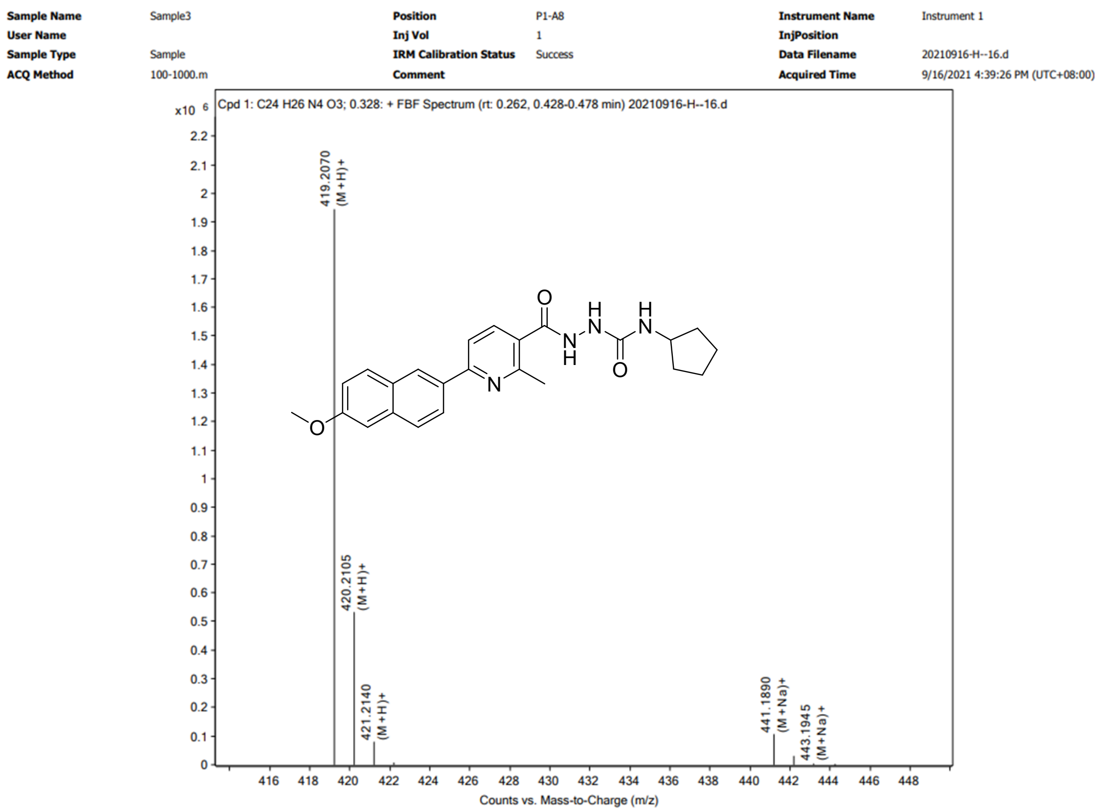

Figure S3. ESI-HRMS of compound **9a.**

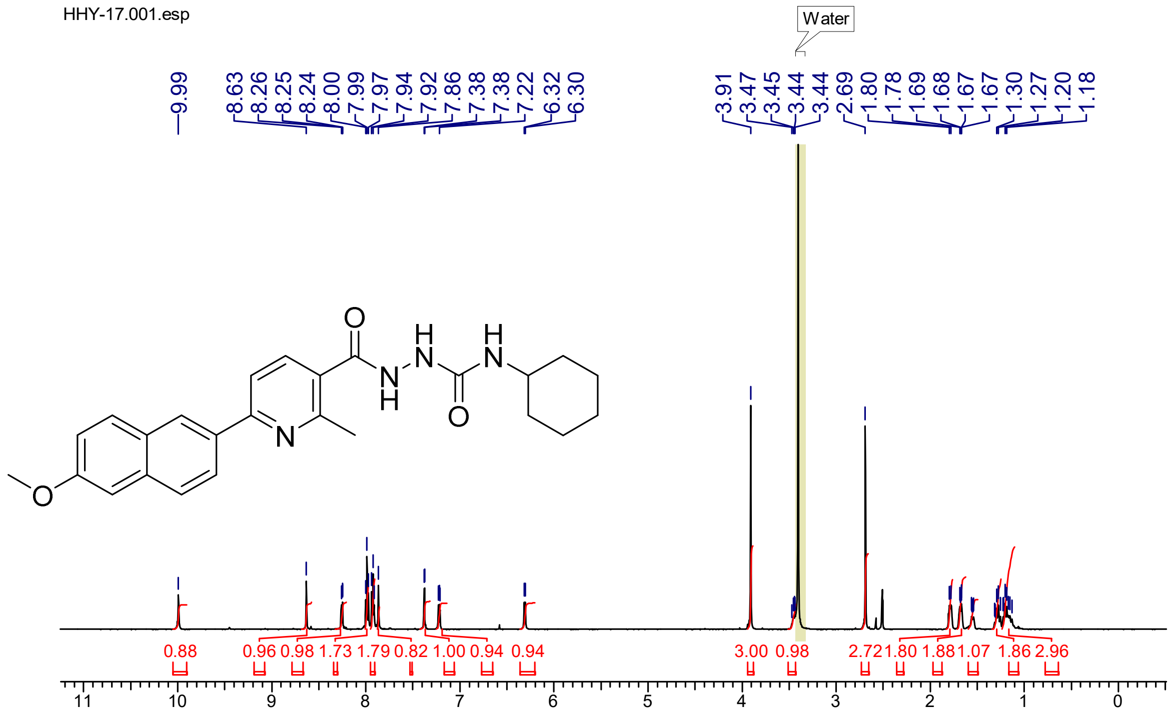

Figure S4. ^{1}H-NMR Spectrum of compound **9b.**

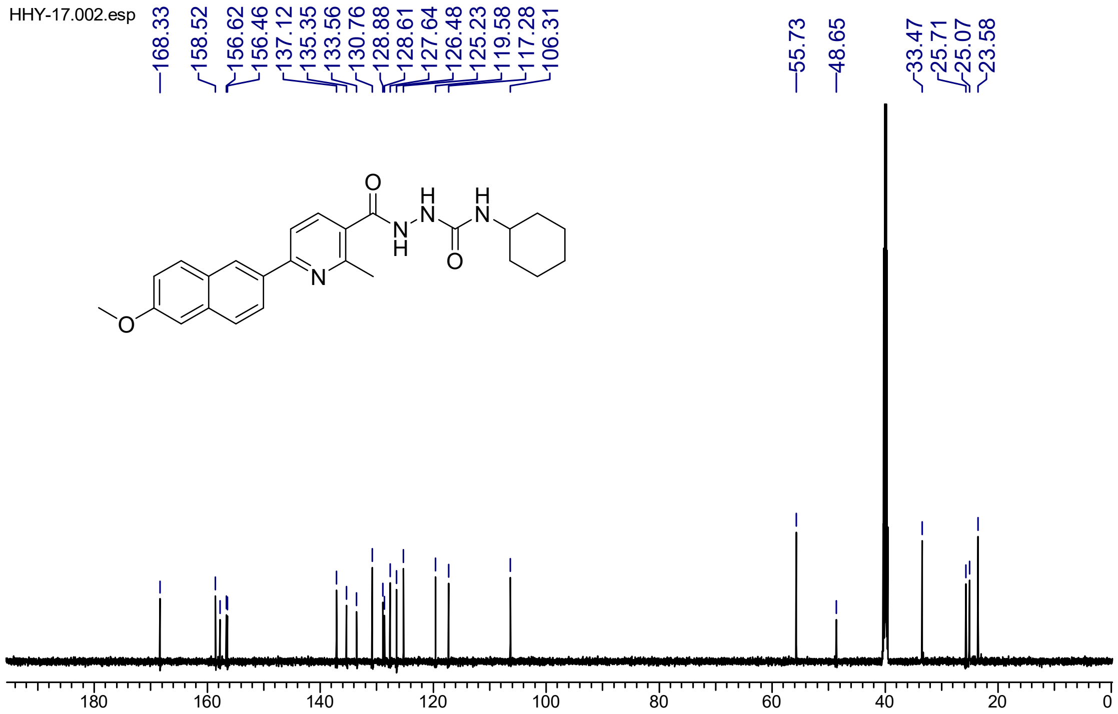

Figure S5. ^{1}C-NMR Spectrum of compound **9b.**

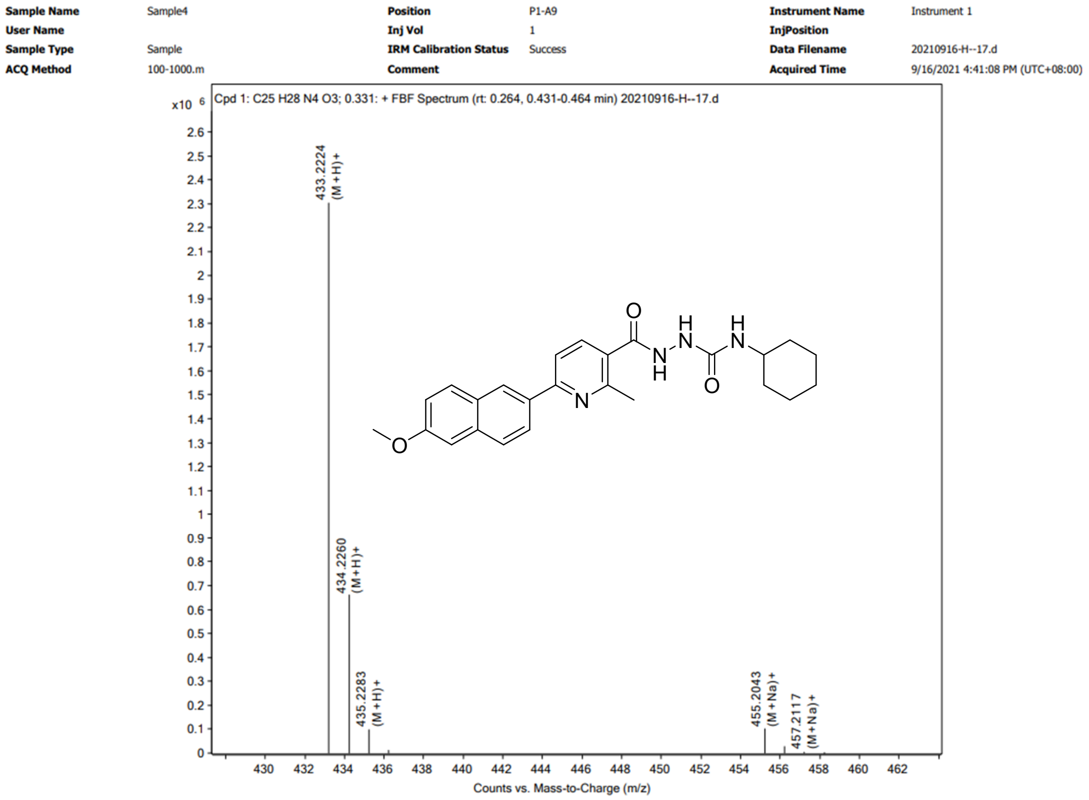

Figure S6. ESI-HRMS of compound **9b.**

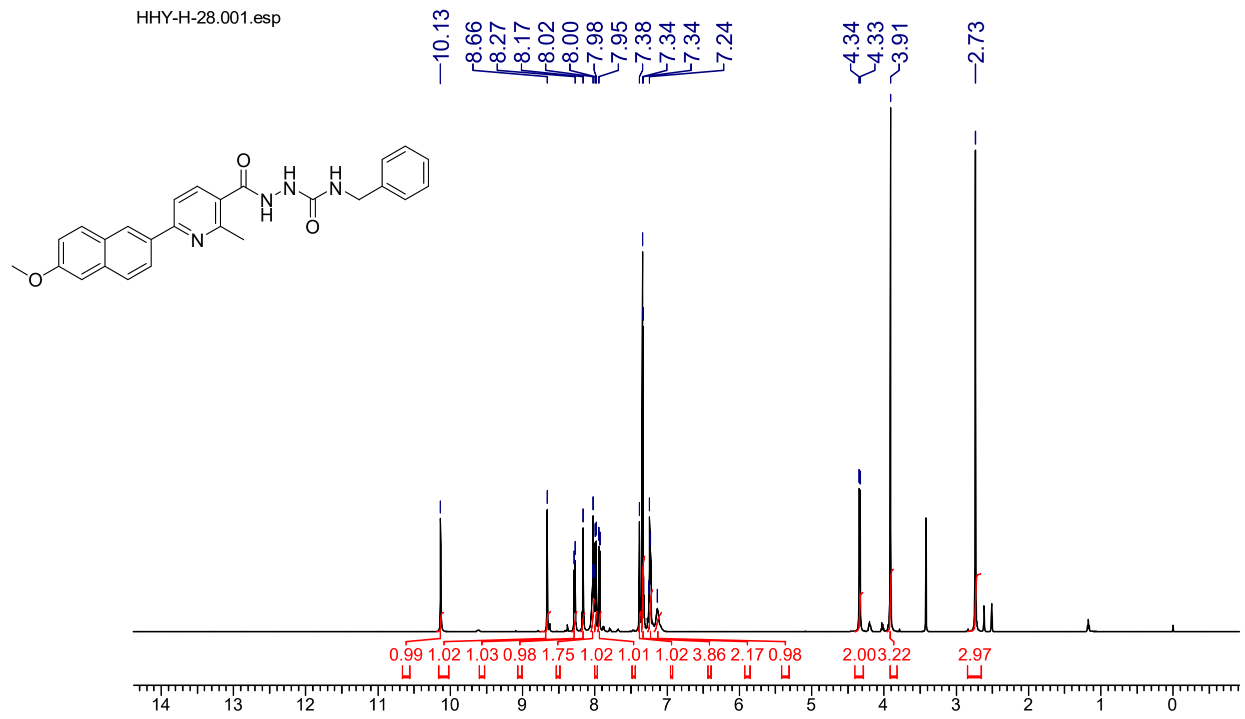

Figure S7. ^{1}H-NMR Spectrum of compound **9c.**

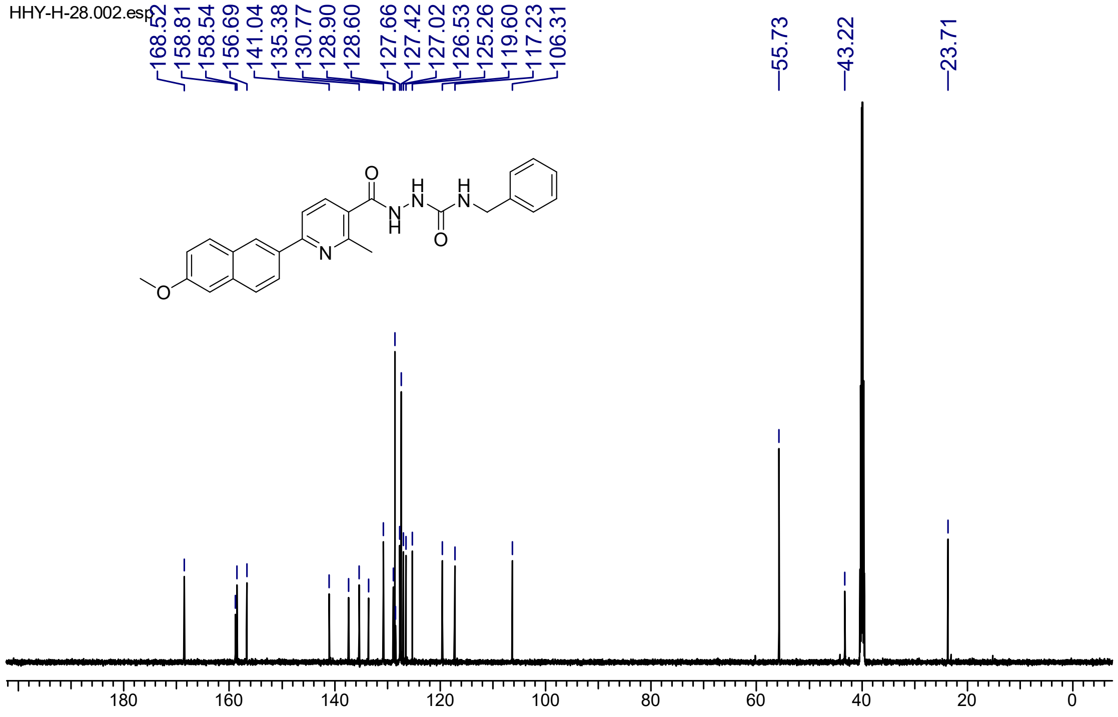

Figure S8. [1]C-NMR Spectrum of compound **9c.**

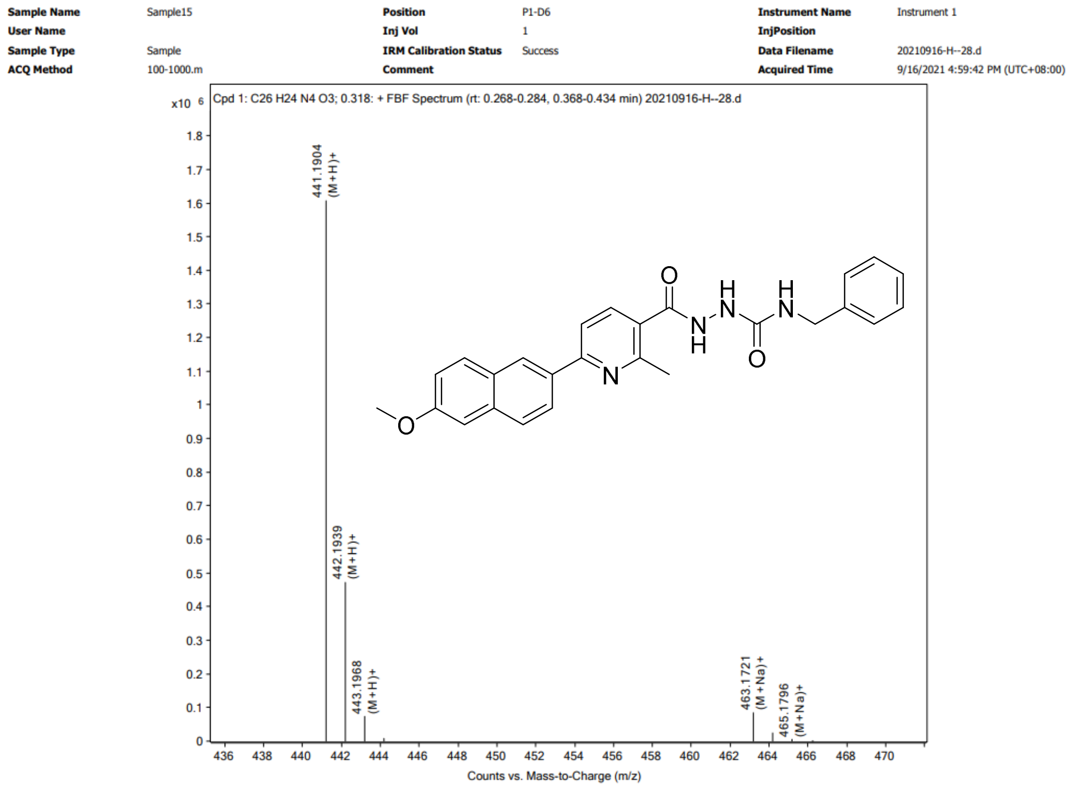

Figure S9. ESI-HRMS of compound **9c.**

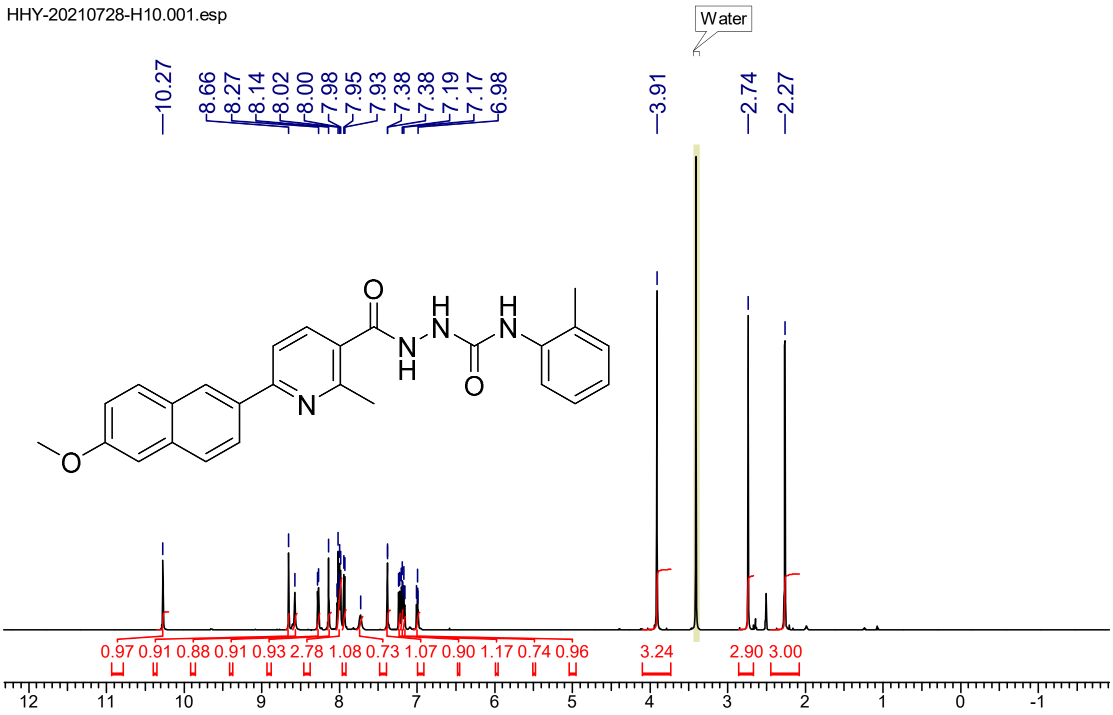

Figure S10. ^{1}H-NMR Spectrum of compound **9d.**

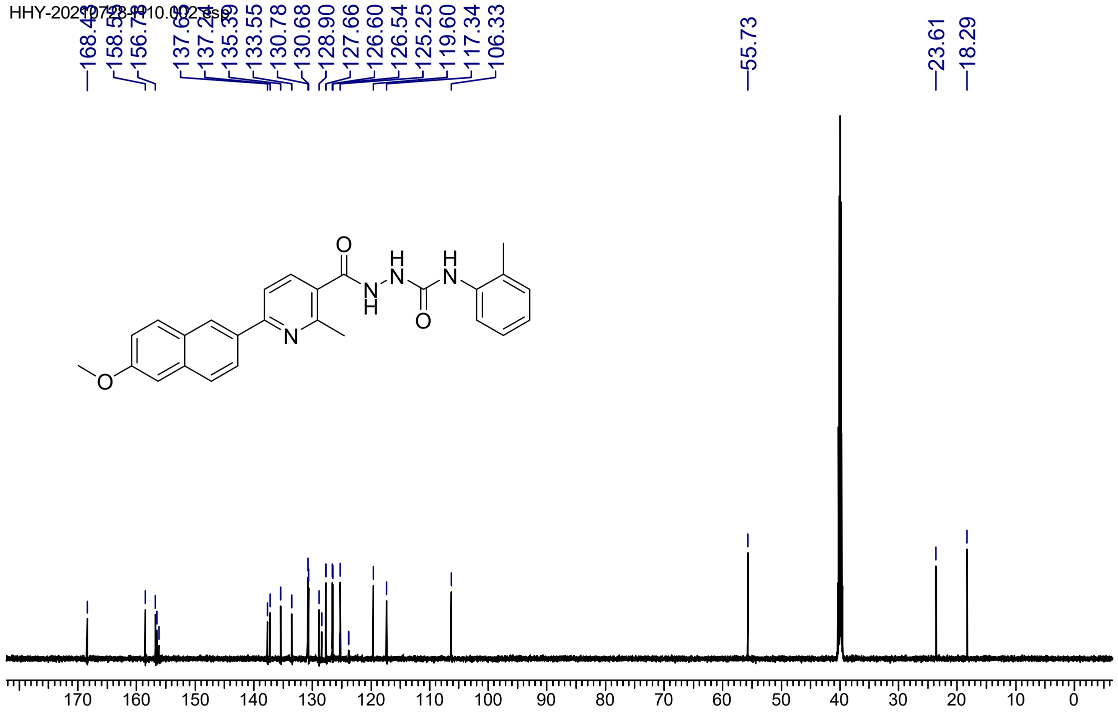

Figure S11. [1]C-NMR Spectrum of compound **9d.**

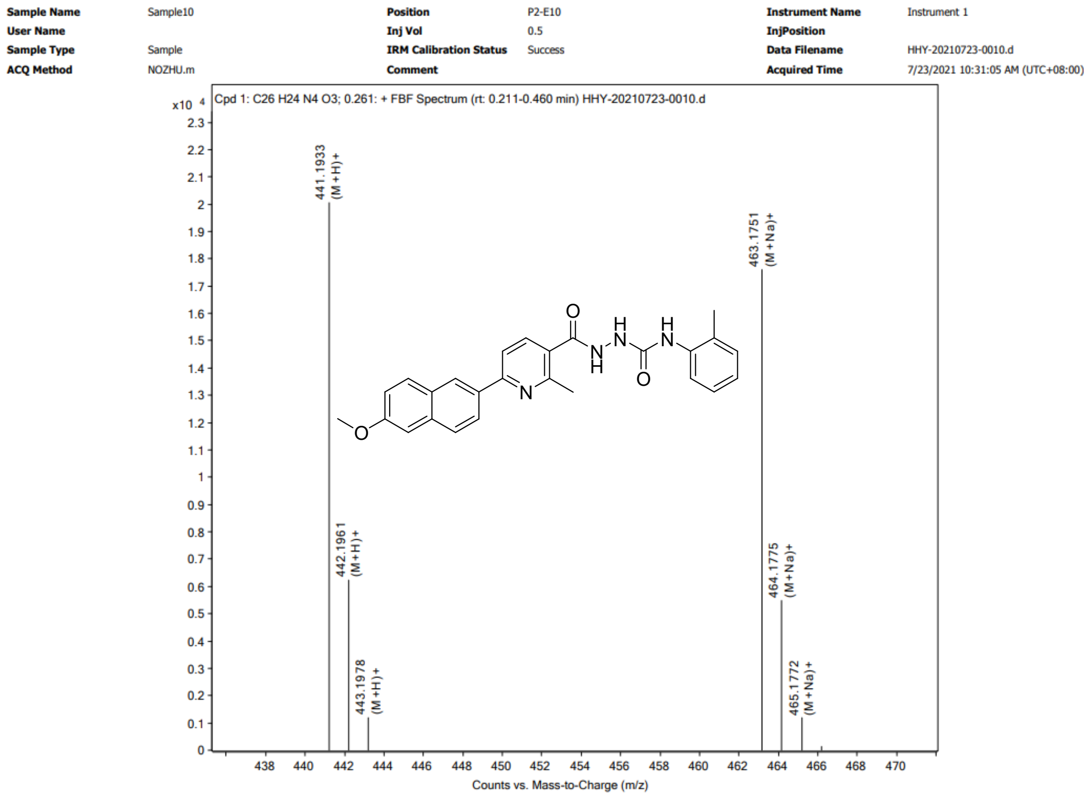

Figure S12. ESI-HRMS of compound **9d.**

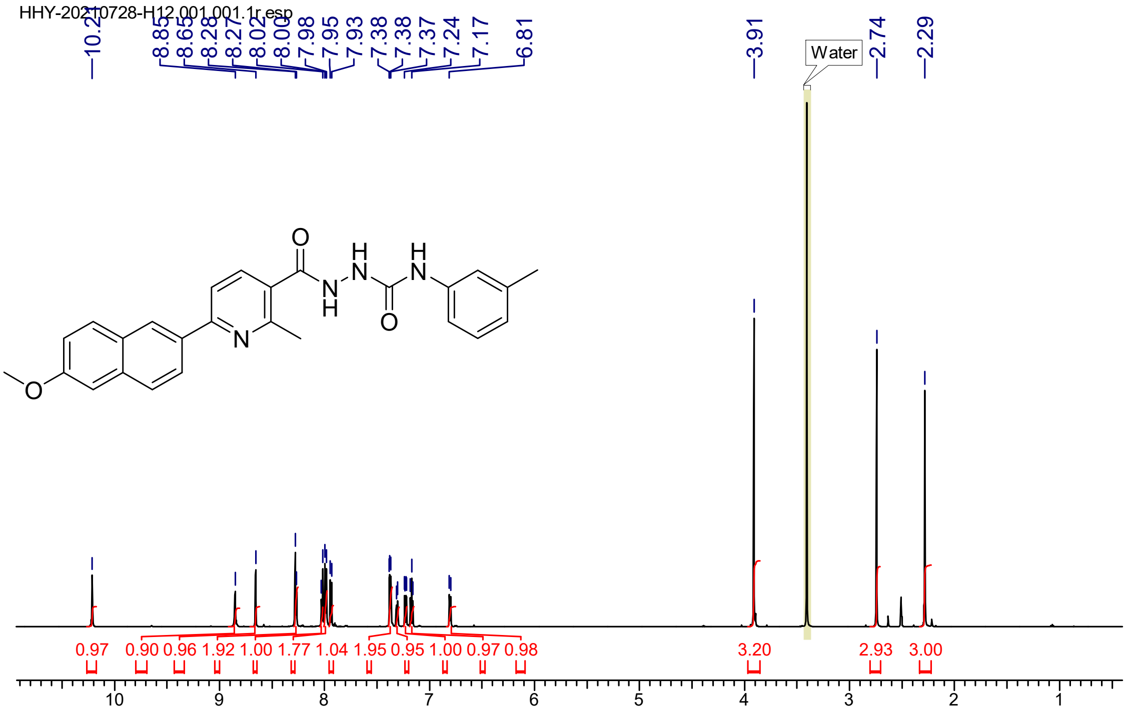

Figure S13. ^{1}H-NMR Spectrum of compound **9e.**

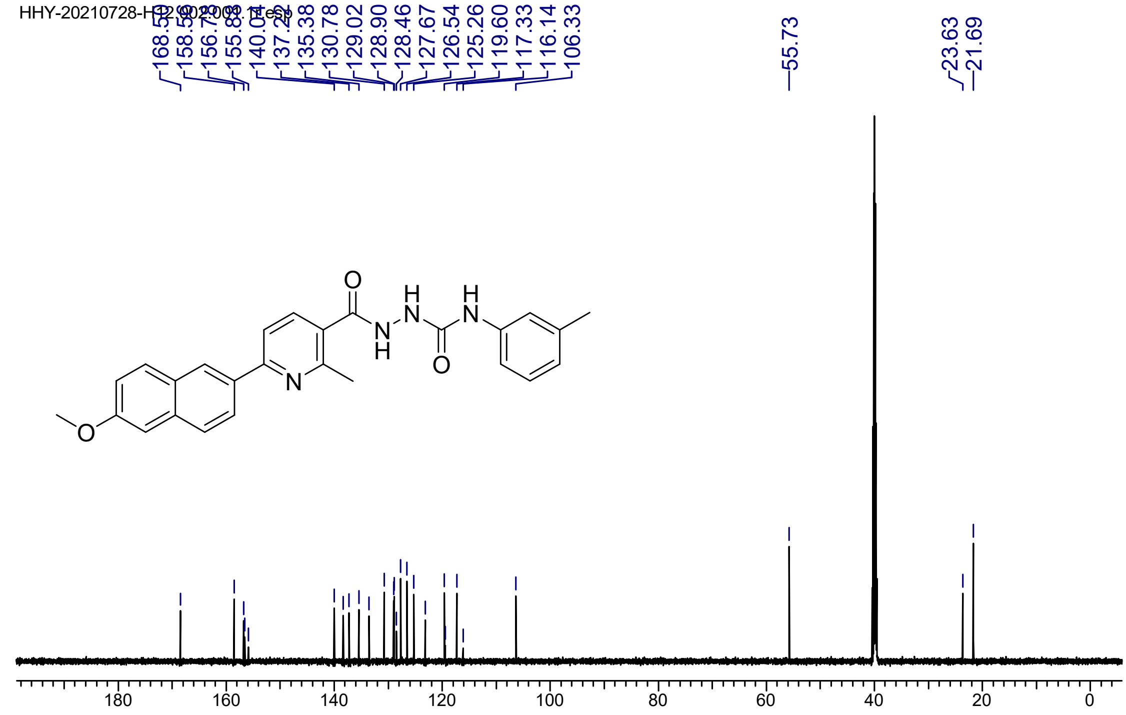

Figure S14. [1]C-NMR Spectrum of compound **9e.**

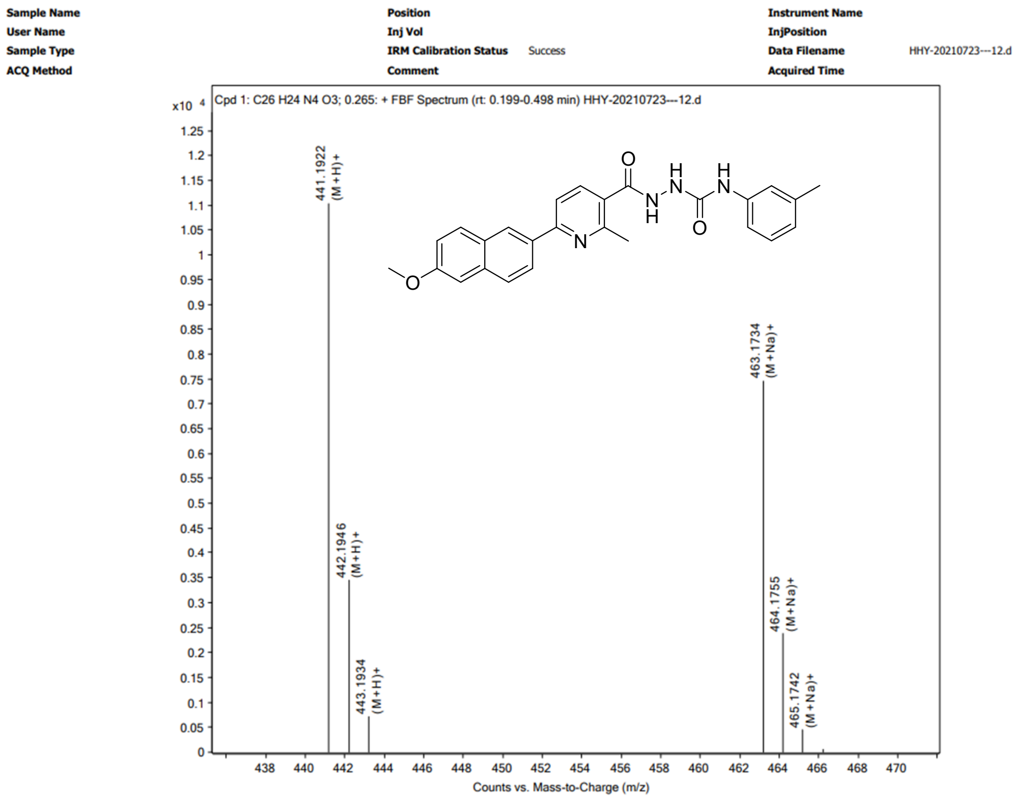

Figure S15. ESI-HRMS of compound **9e.**

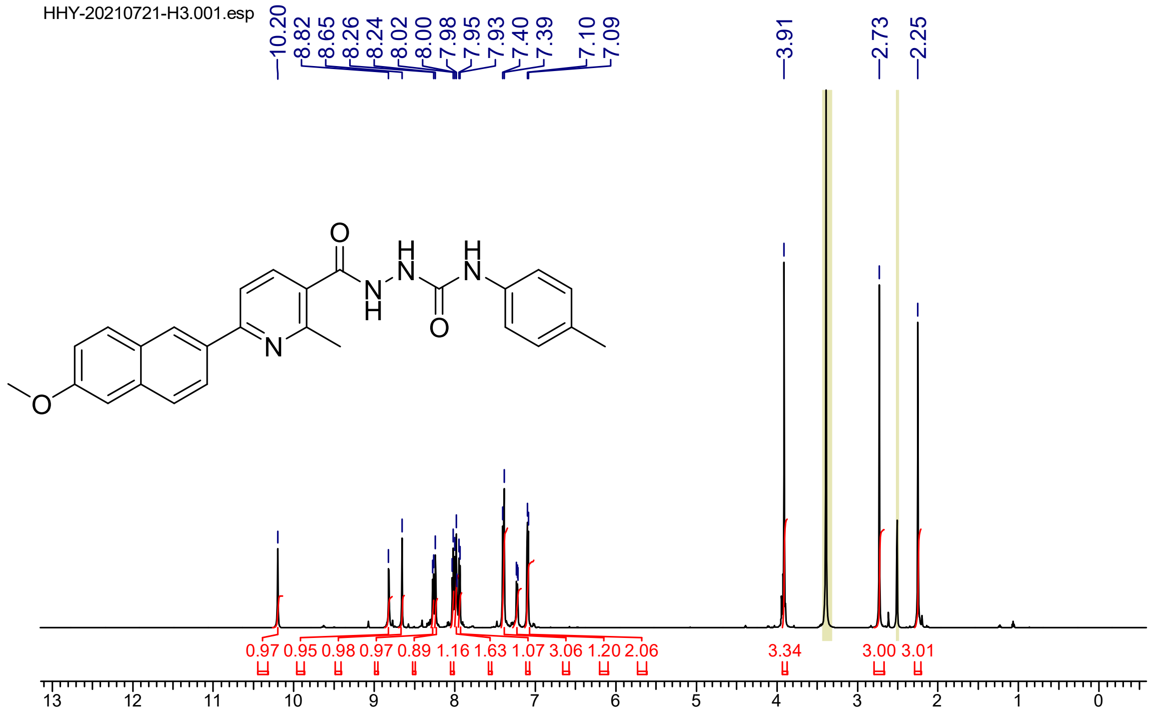

Figure S16. ^{1}H-NMR Spectrum of compound **9f.**

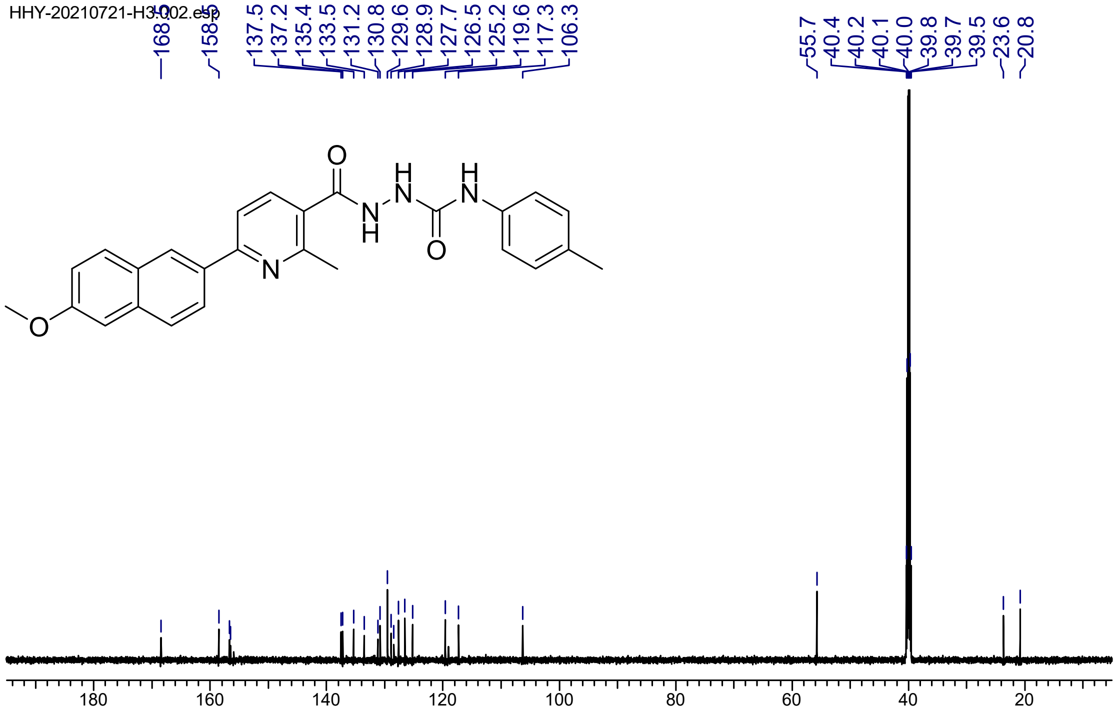

Figure S17. [1]C-NMR Spectrum of compound **9f.**

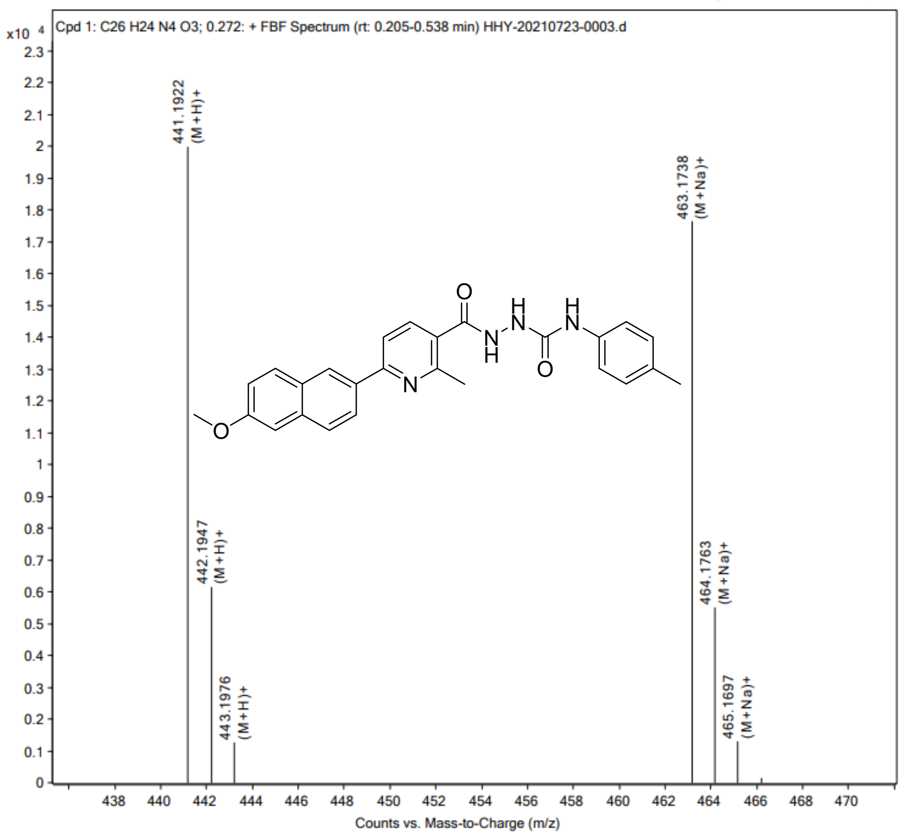

Figure S18. ESI-HRMS of compound **9f.**

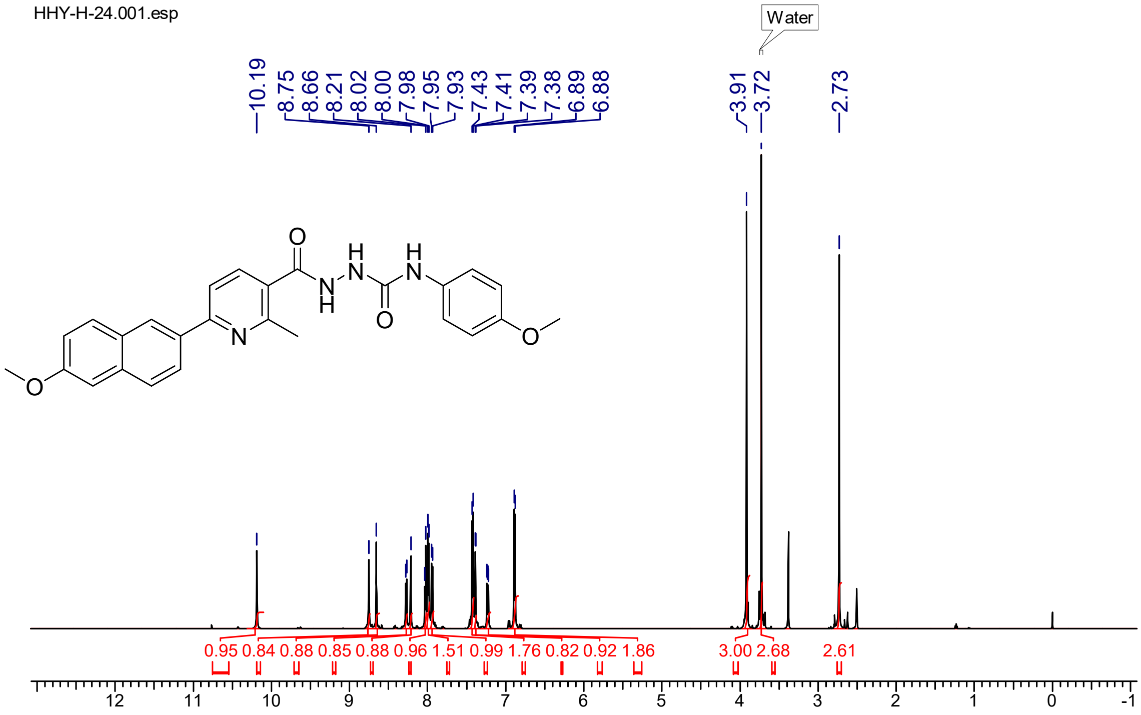

Figure S19. ^{1}H-NMR Spectrum of compound **9g.**

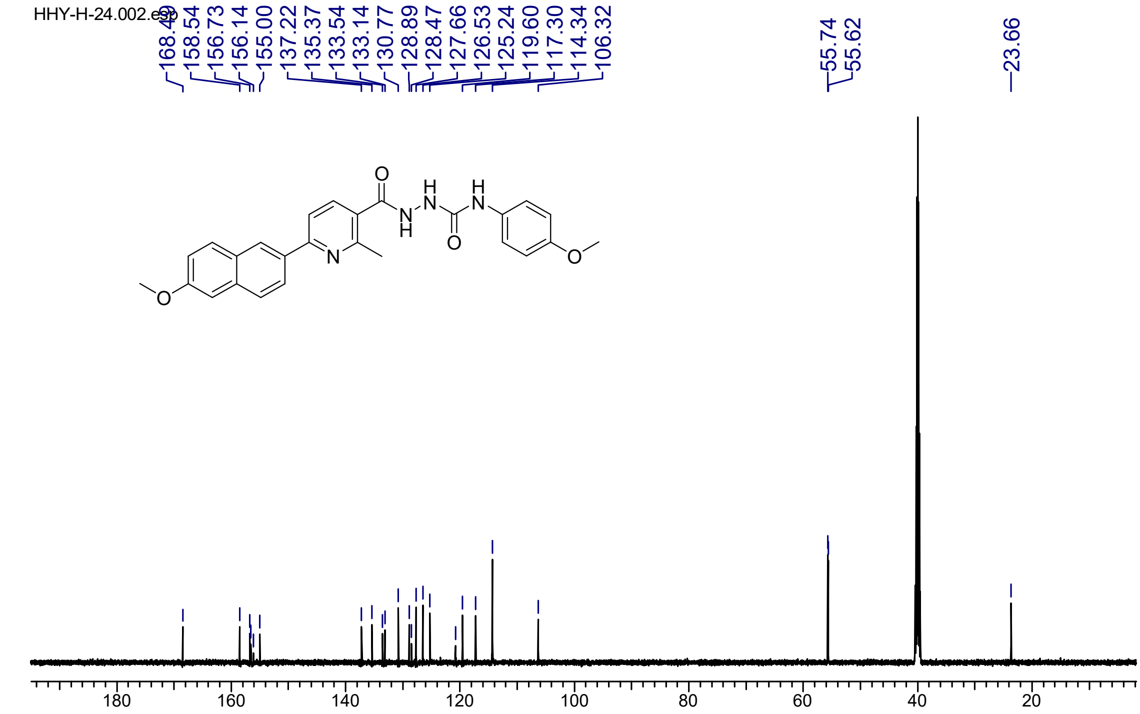

Figure S20. [1]C-NMR Spectrum of compound **9g.**

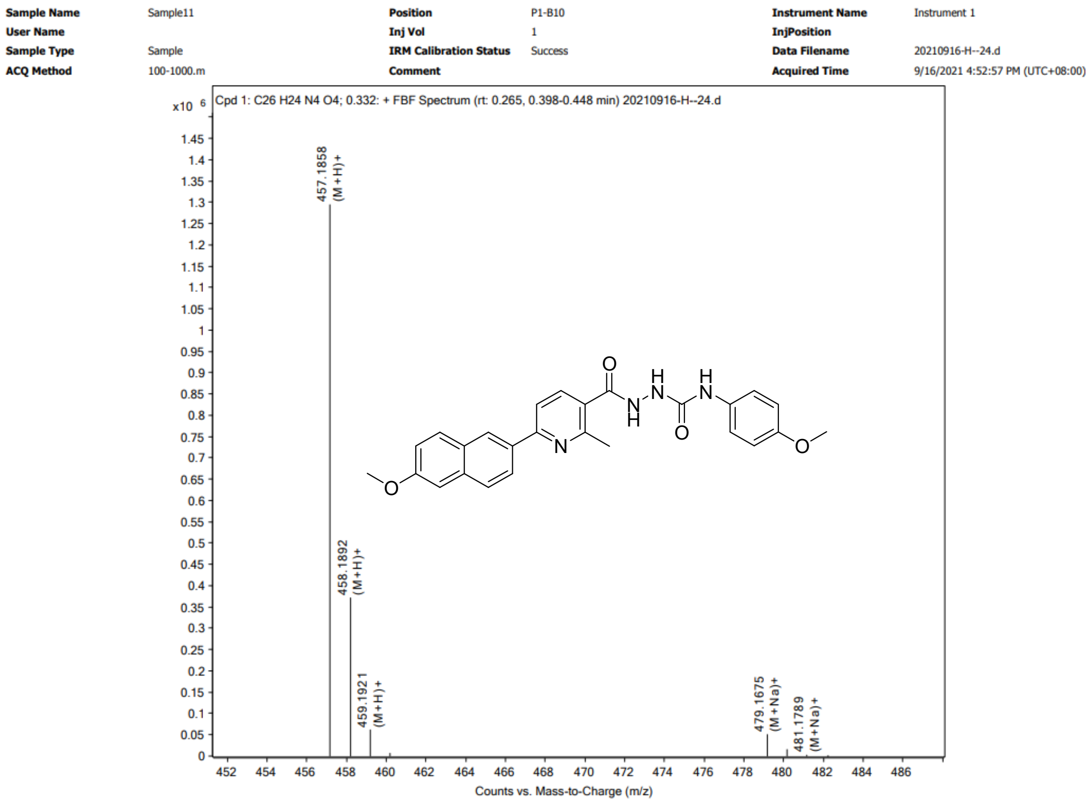

Figure S21. ESI-HRMS of compound **9g.**

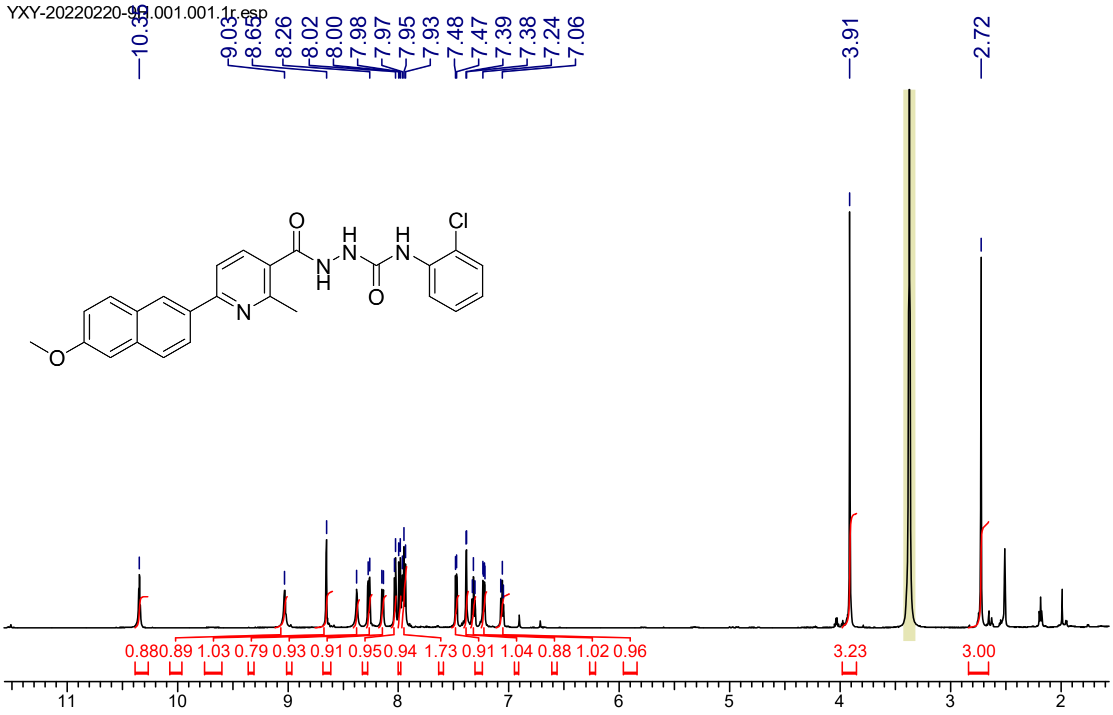

Figure S22. ^{1}H-NMR Spectrum of compound **9h.**

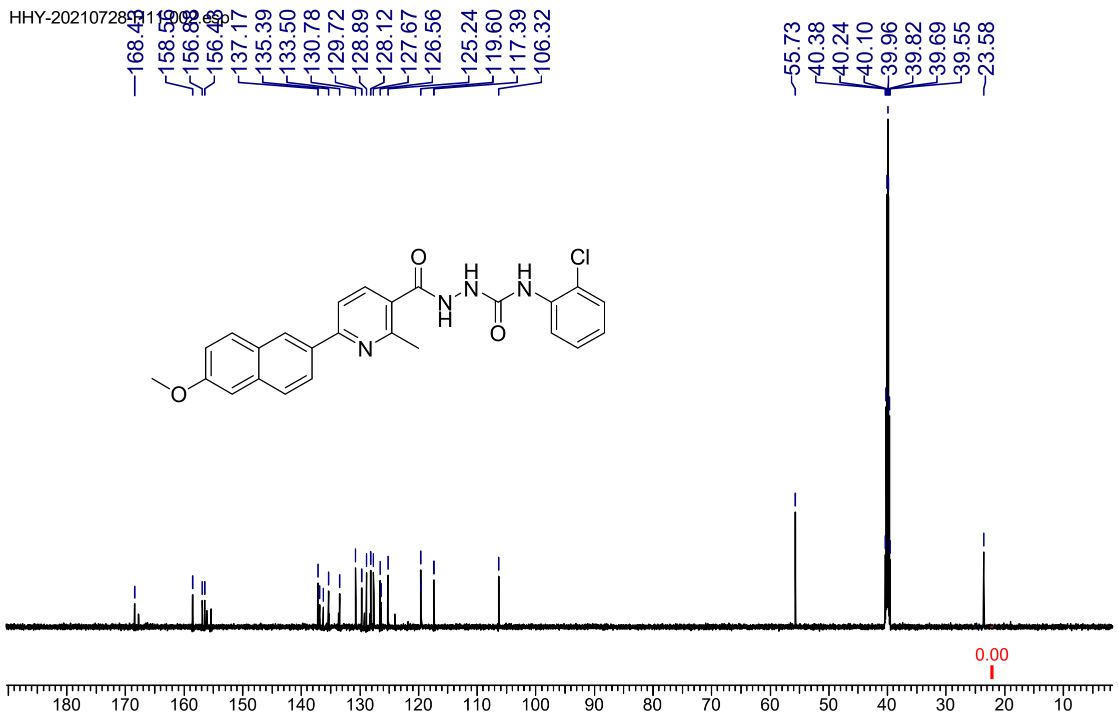

Figure S23. [1]C-NMR Spectrum of compound **9h.**

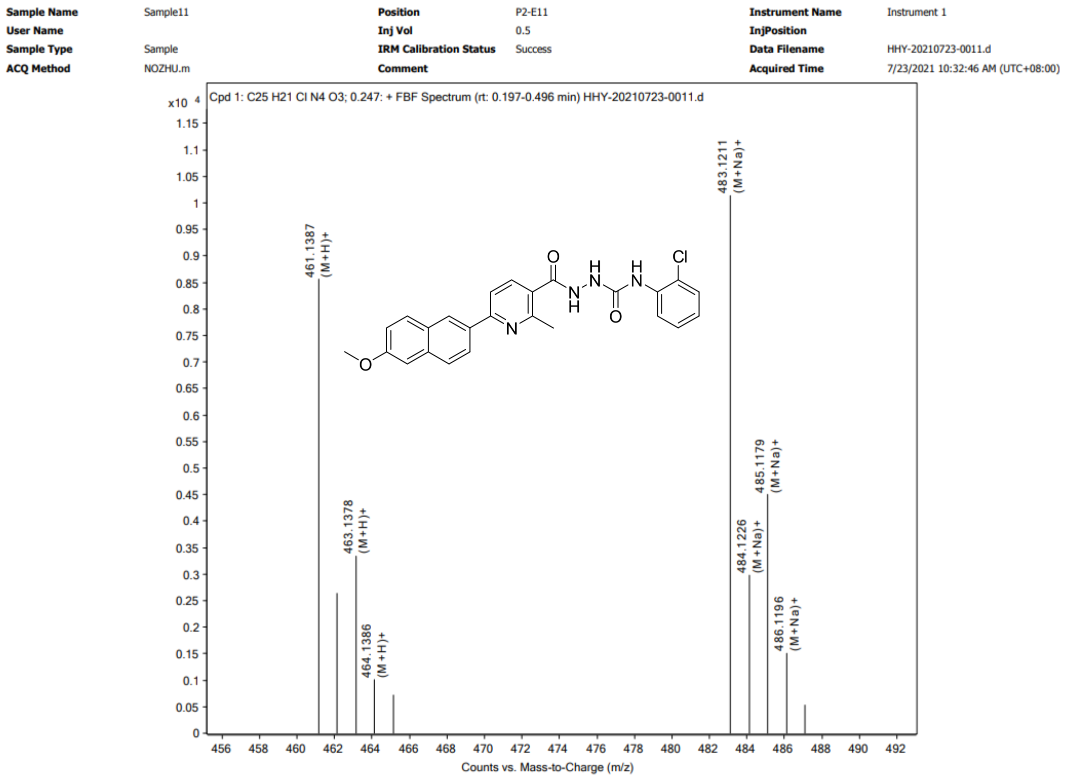

Figure S24. ESI-HRMS of compound **9h.**

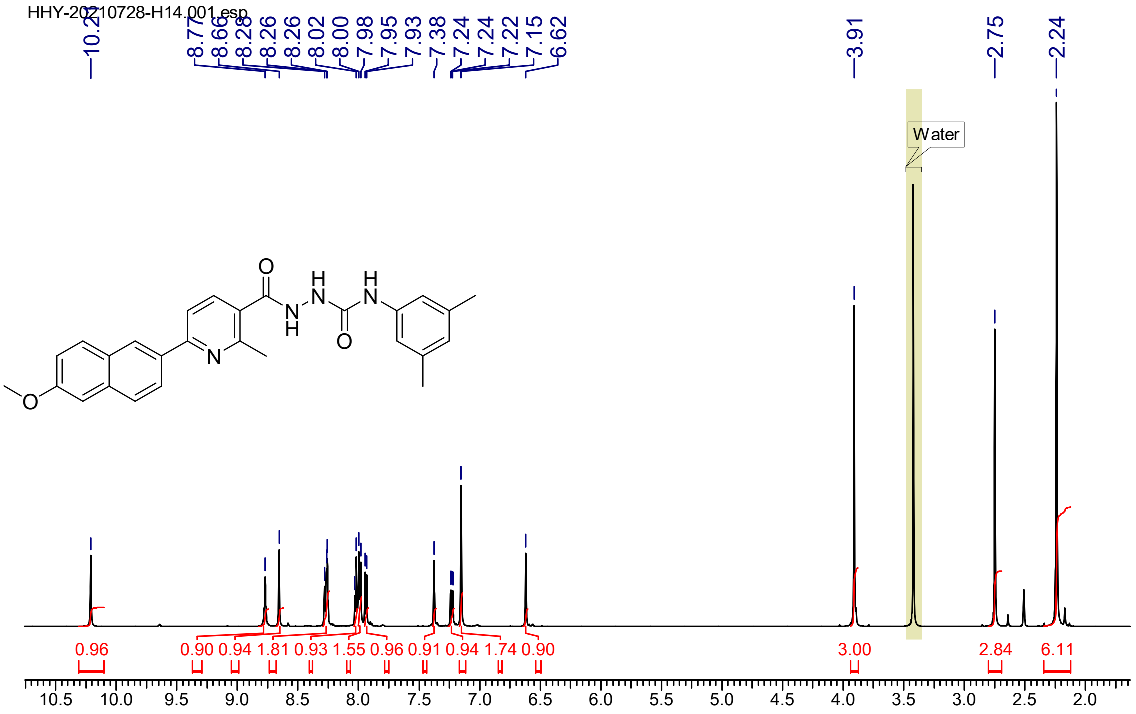

Figure S25. ¹H-NMR Spectrum of compound **9i.**

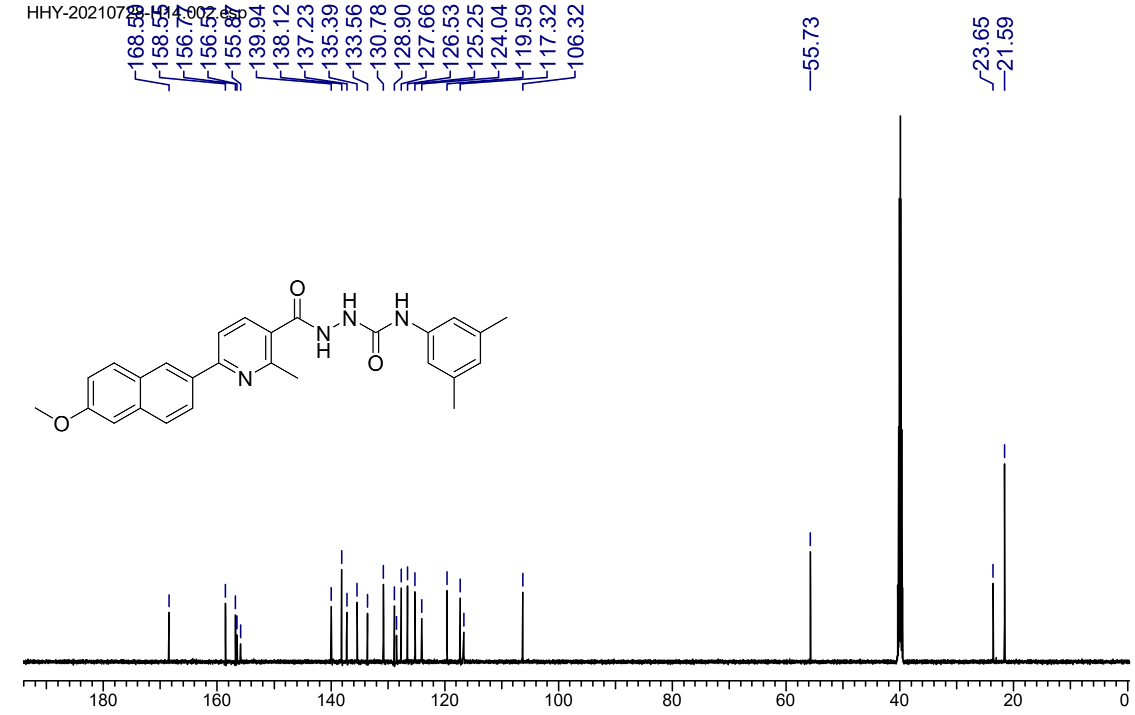

Figure S26. ¹C-NMR Spectrum of compound **9i.**

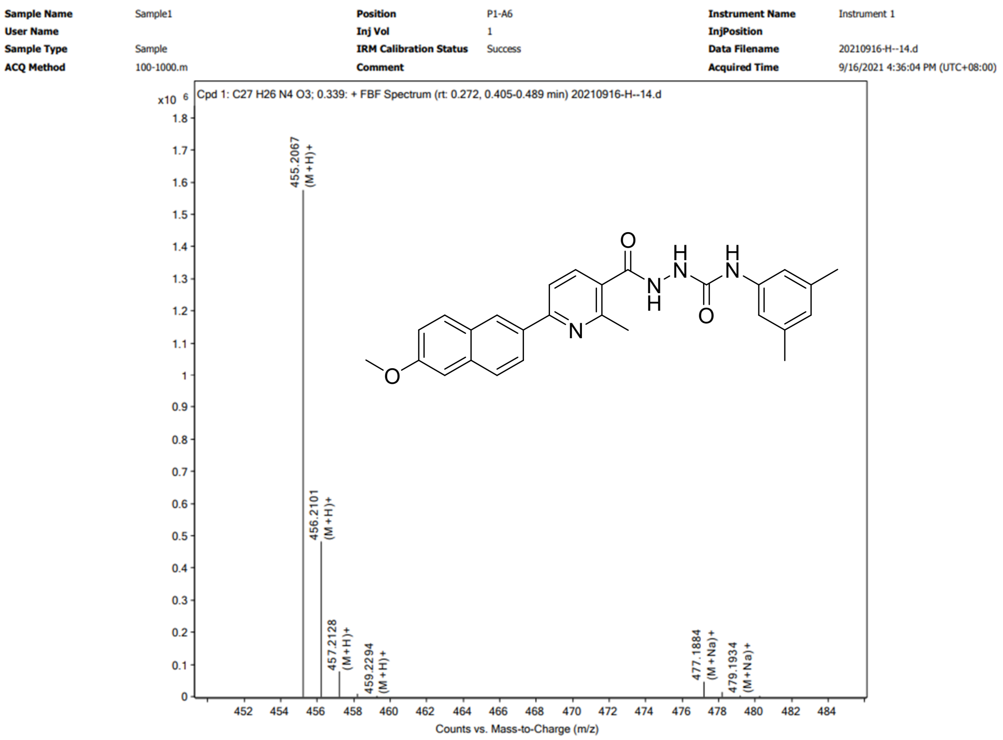

Figure S27. ESI-HRMS of compound **9i.**

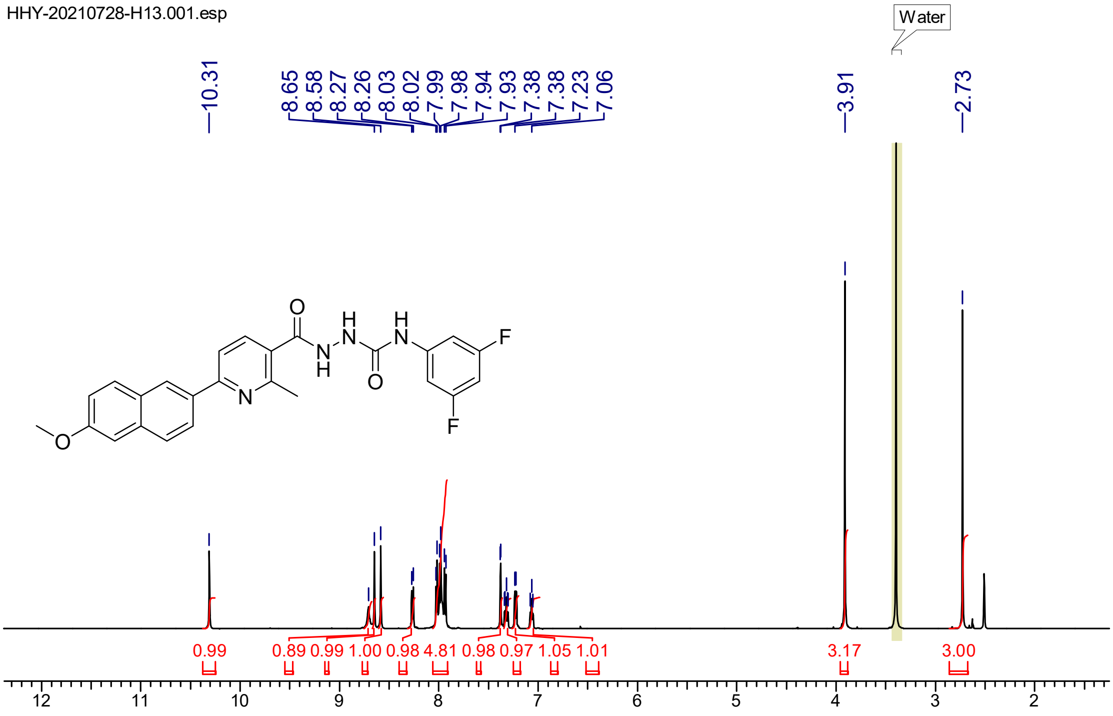

Figure S28. ^{1}H-NMR Spectrum of compound **9j.**

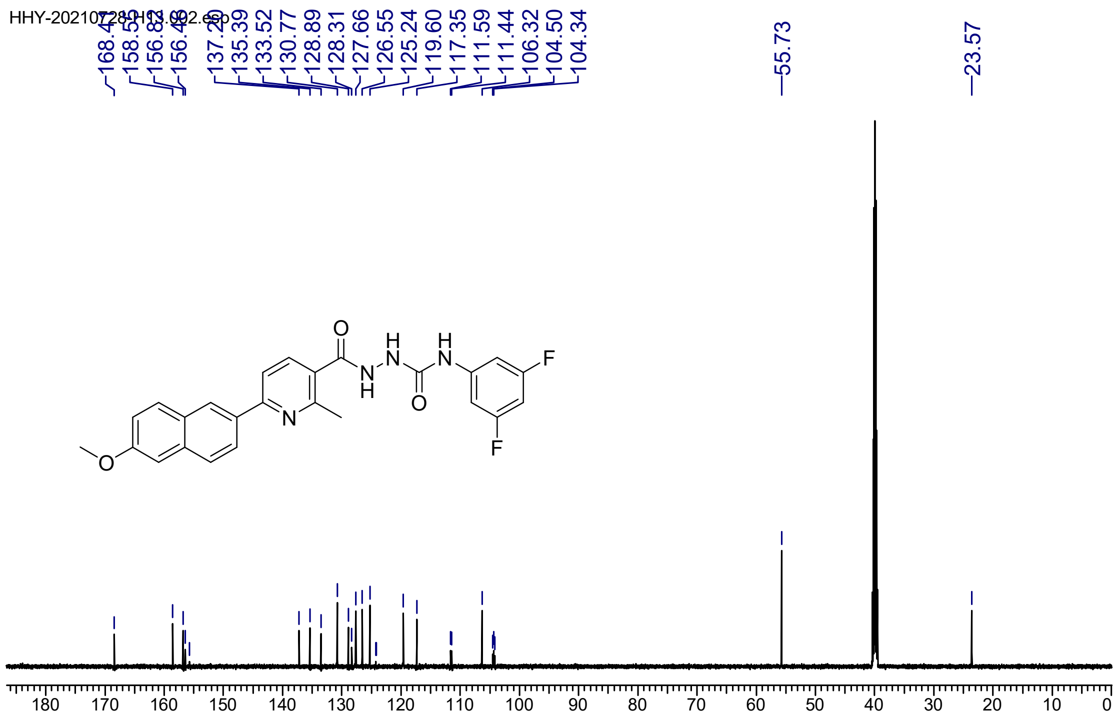

Figure S29. ^{1}C-NMR Spectrum of compound **9j.**

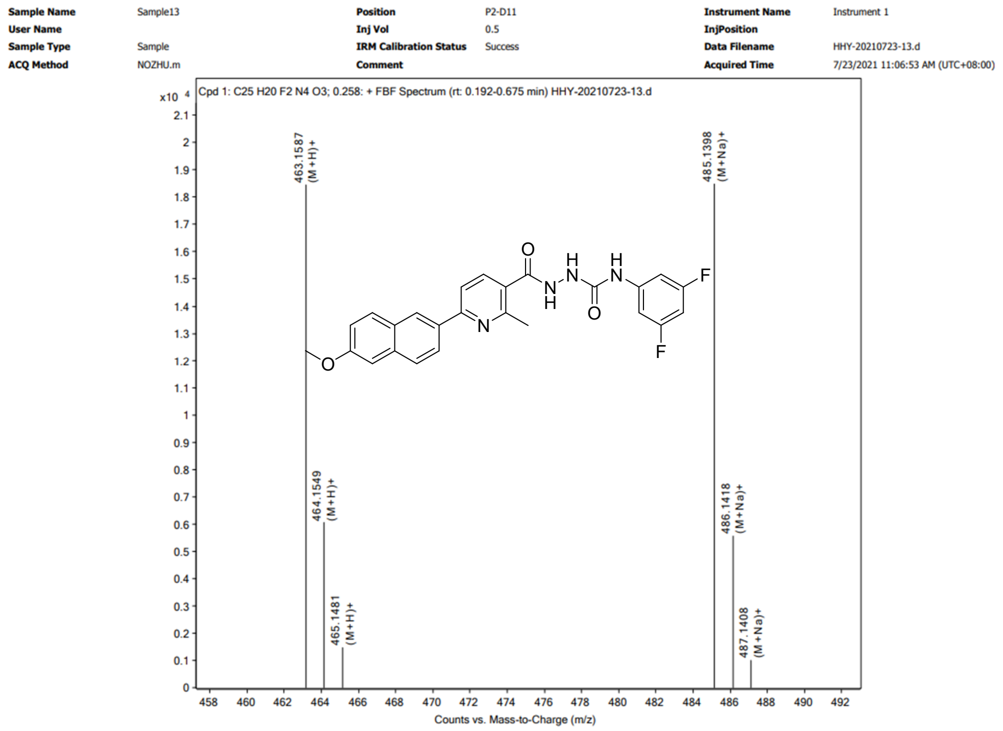

Figure S30. ESI-HRMS of compound **9j.**

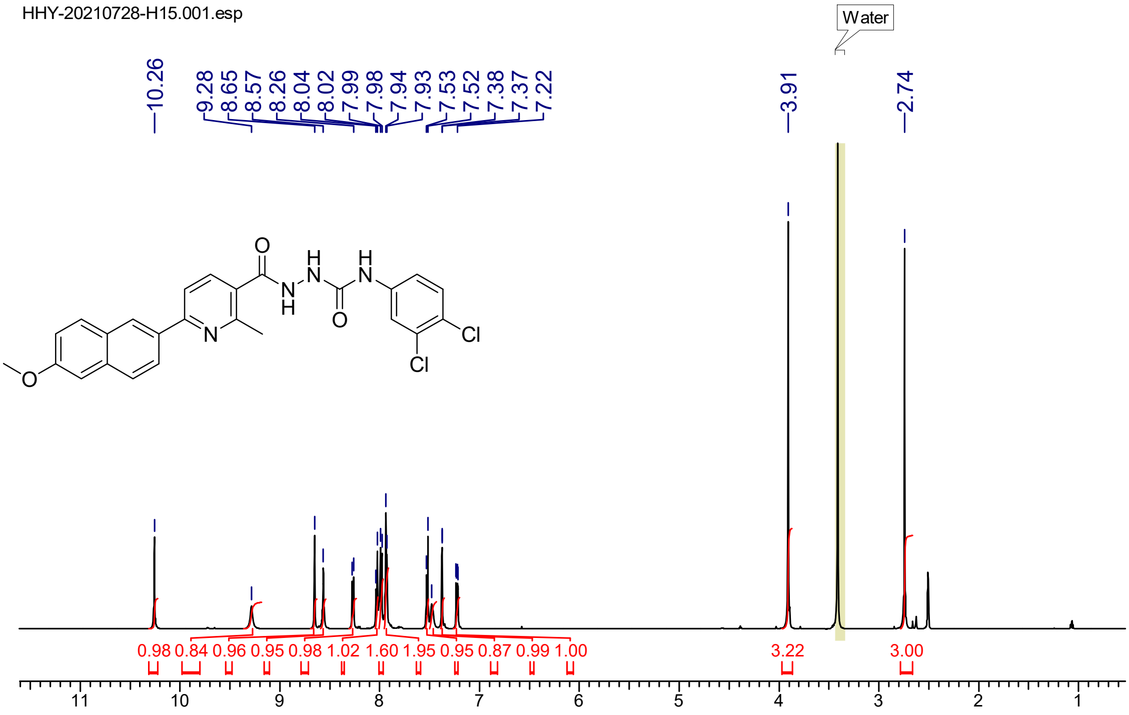

Figure S31. ¹H-NMR Spectrum of compound **9k.**

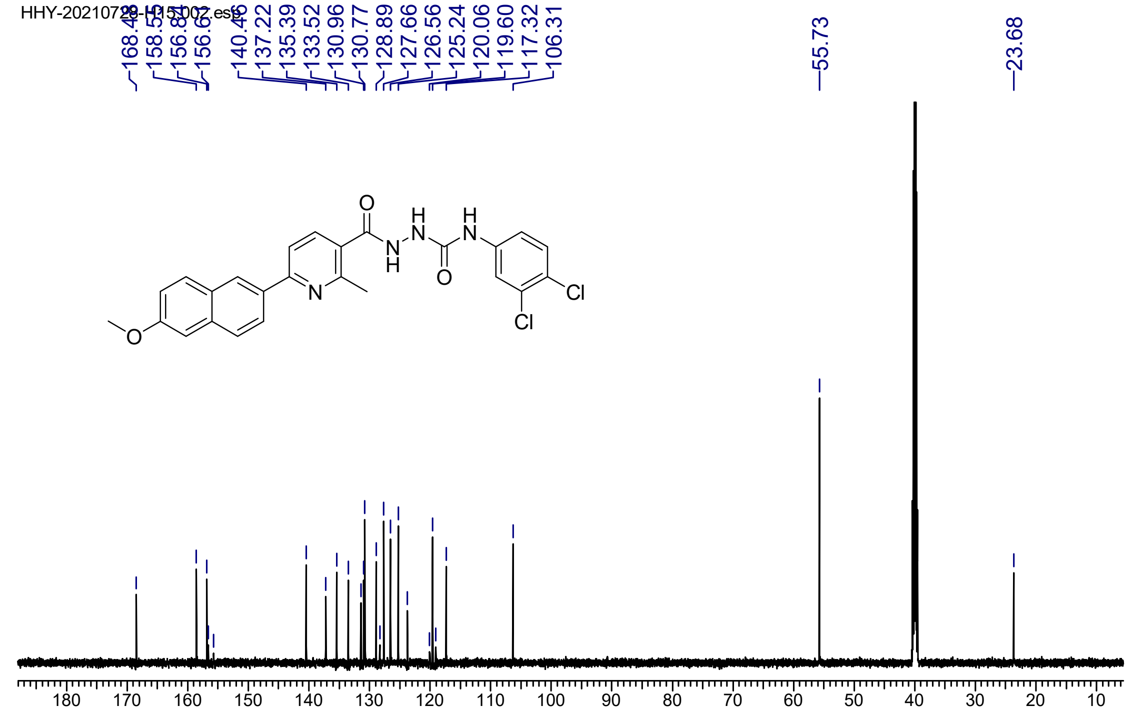

Figure S32. ¹C-NMR Spectrum of compound **9k.**

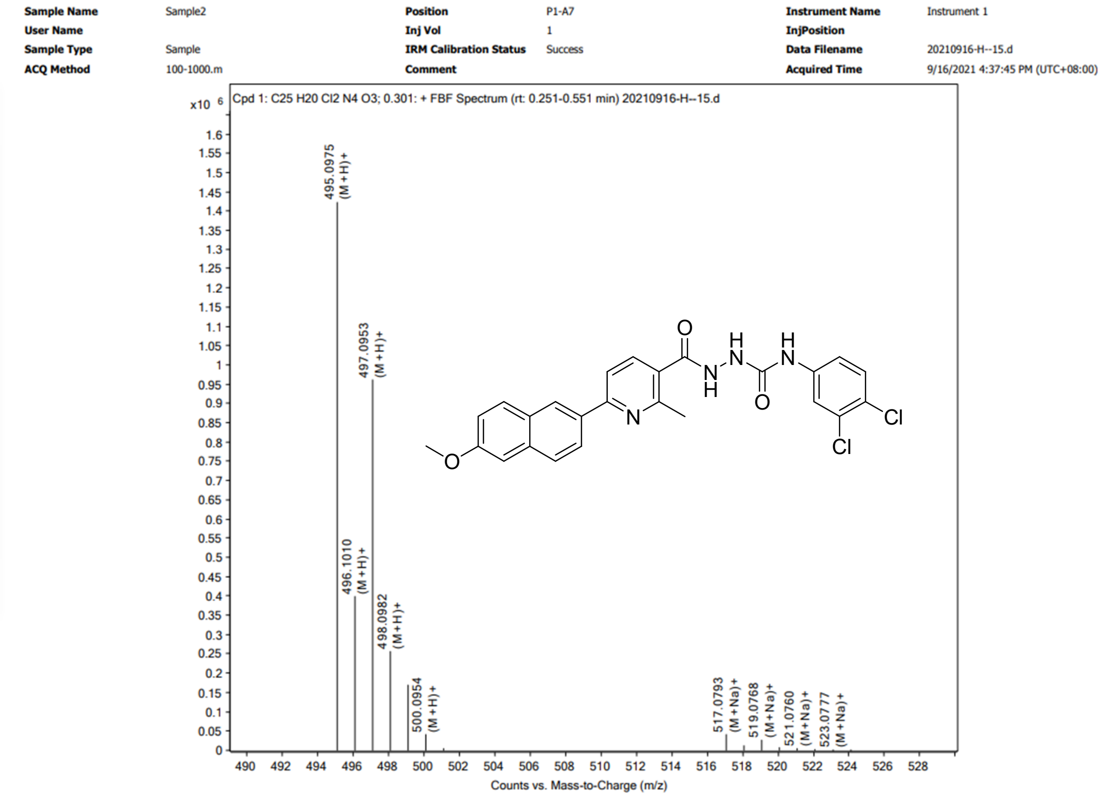

Figure S33. ESI-HRMS of compound **9k.**

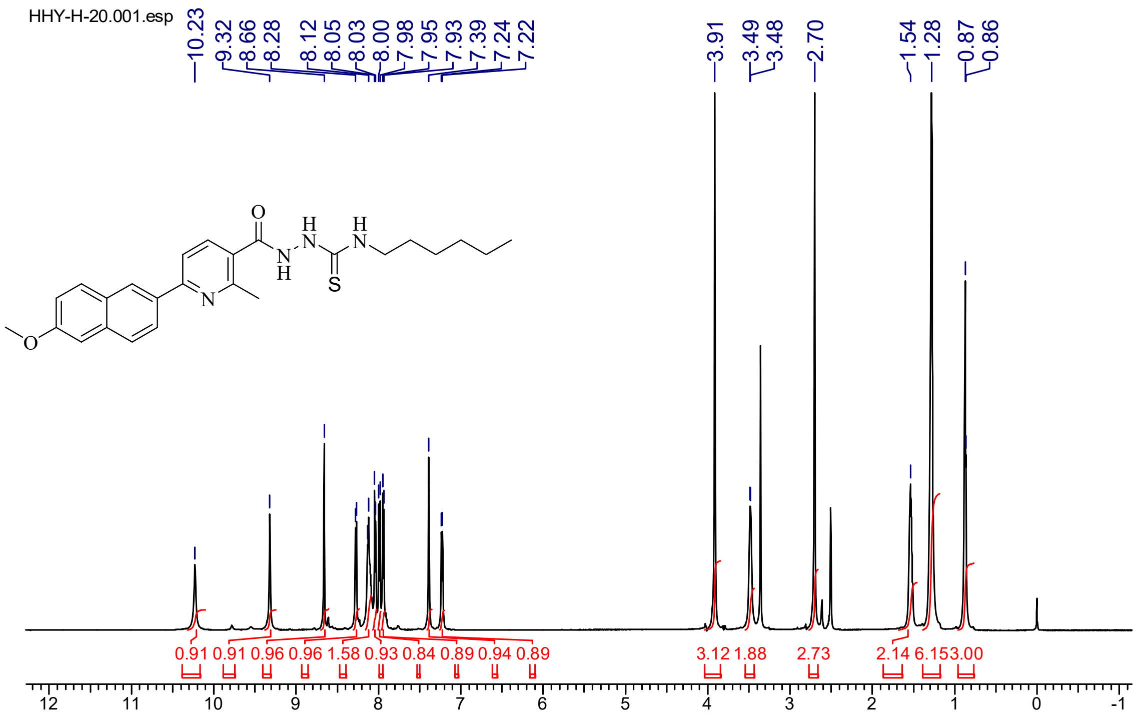

Figure S34. ^{1}H-NMR Spectrum of compound **9l.**

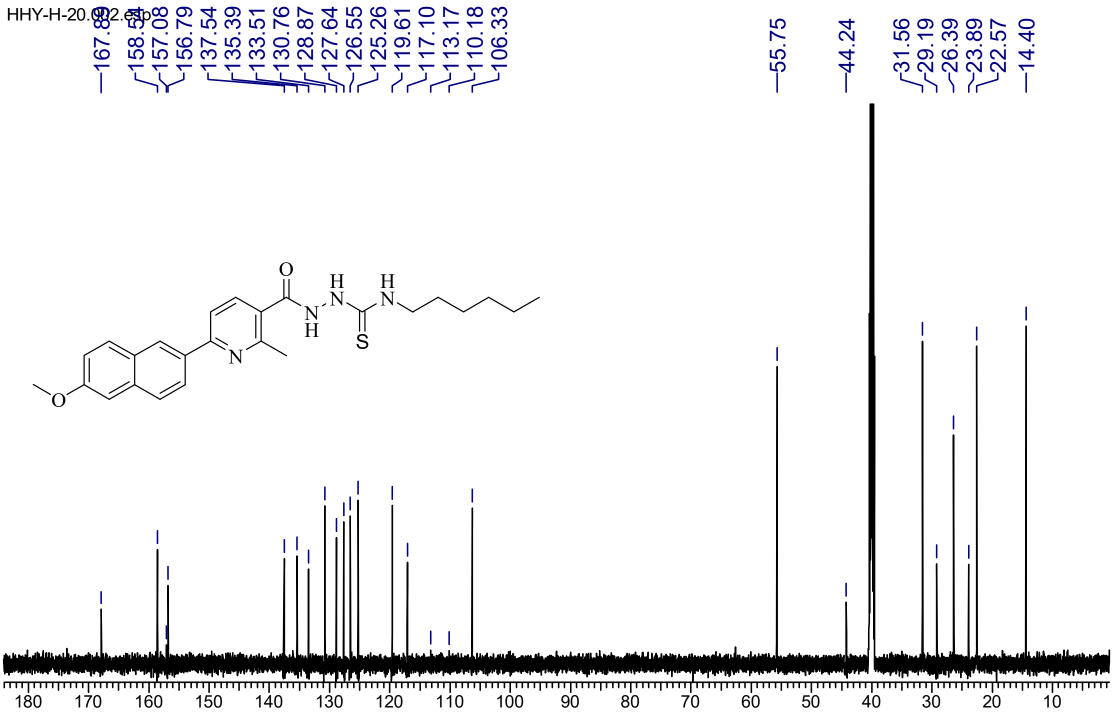

Figure S35. ¹C-NMR Spectrum of compound **9l.**

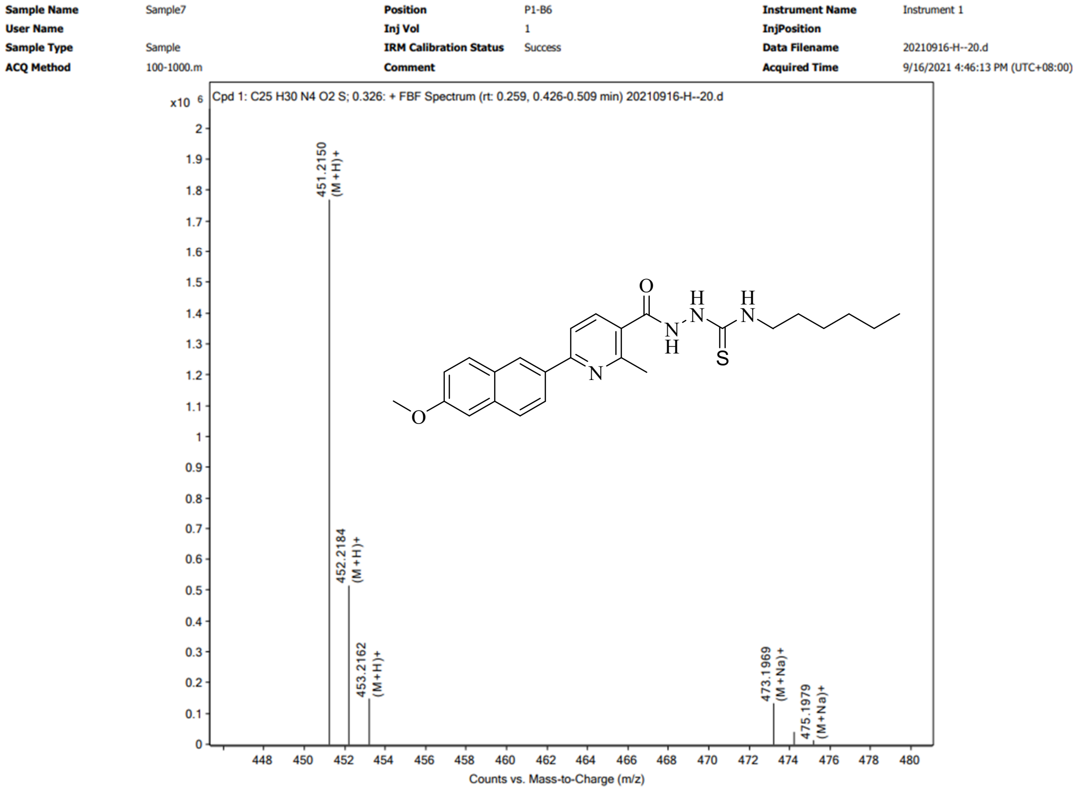

Figure S36. ESI-HRMS of compound **9l.**

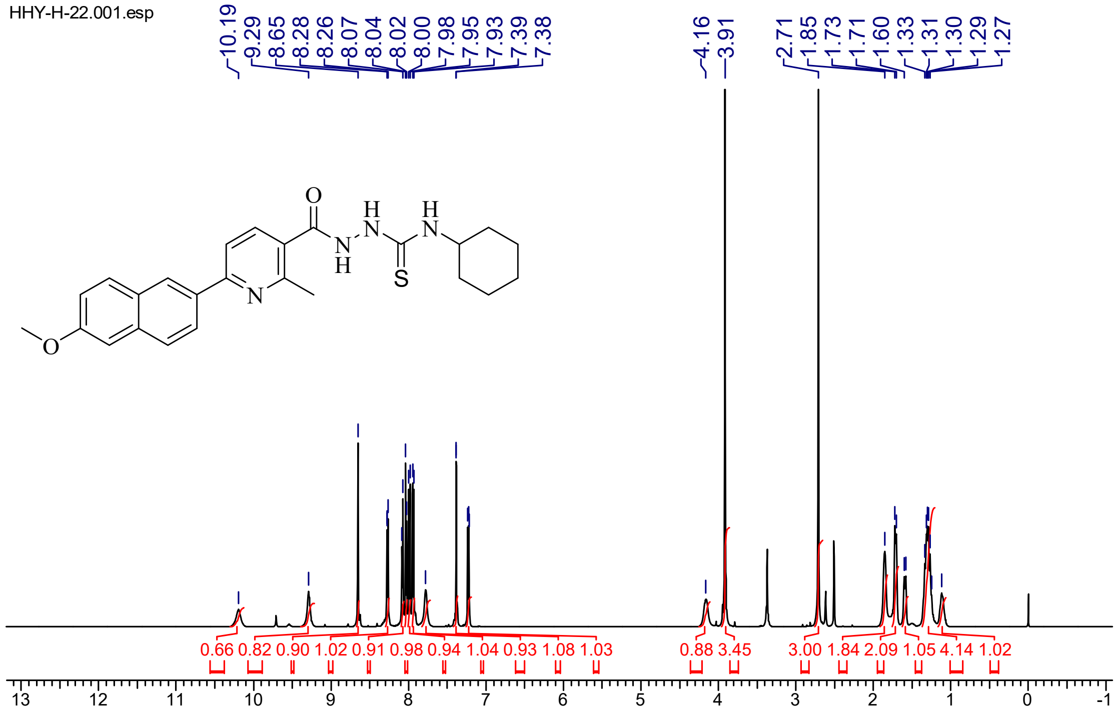

Figure S37. ^{1}H-NMR Spectrum of compound **9m.**

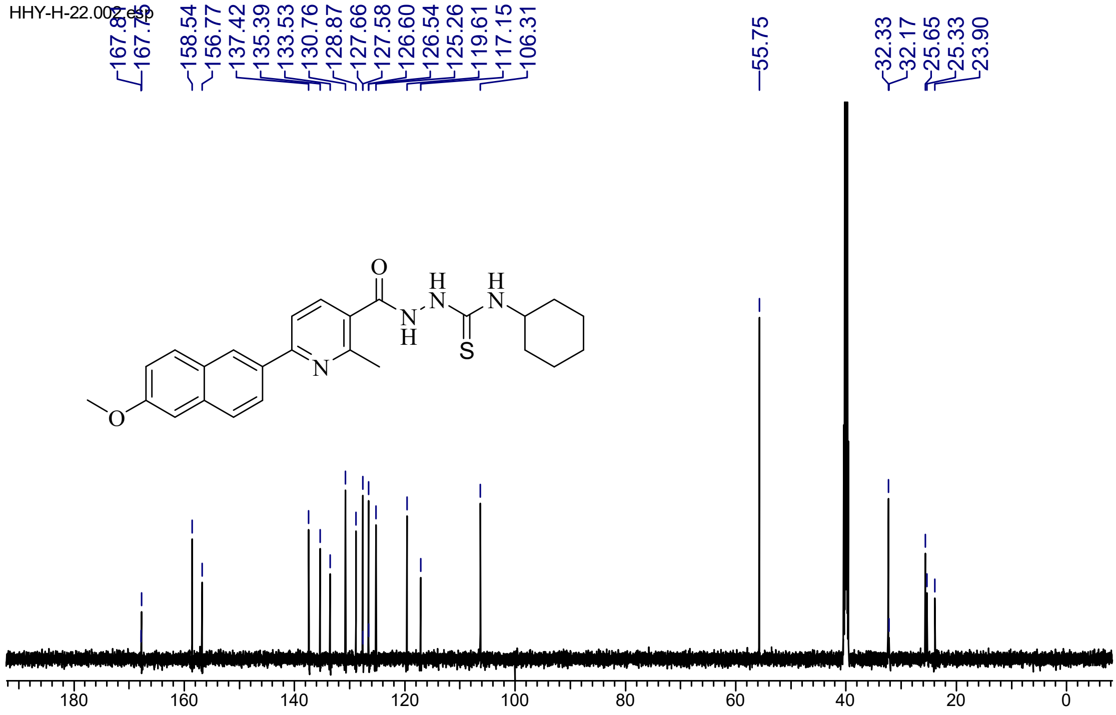

Figure S38. ^{1}C-NMR Spectrum of compound **9m.**

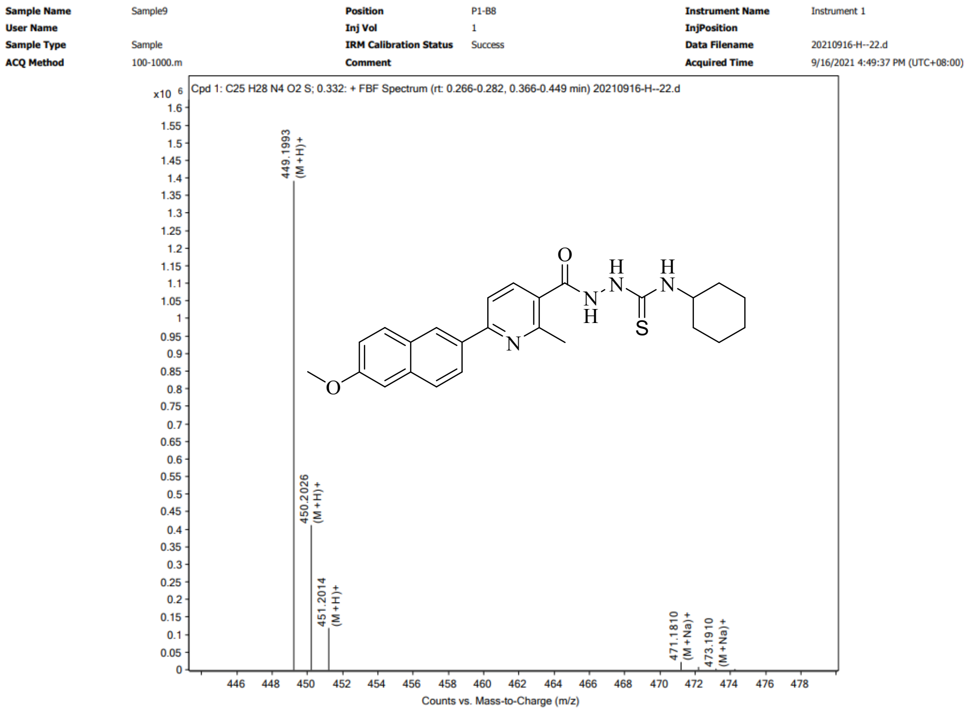

Figure S39. ESI-HRMS of compound **9m.**

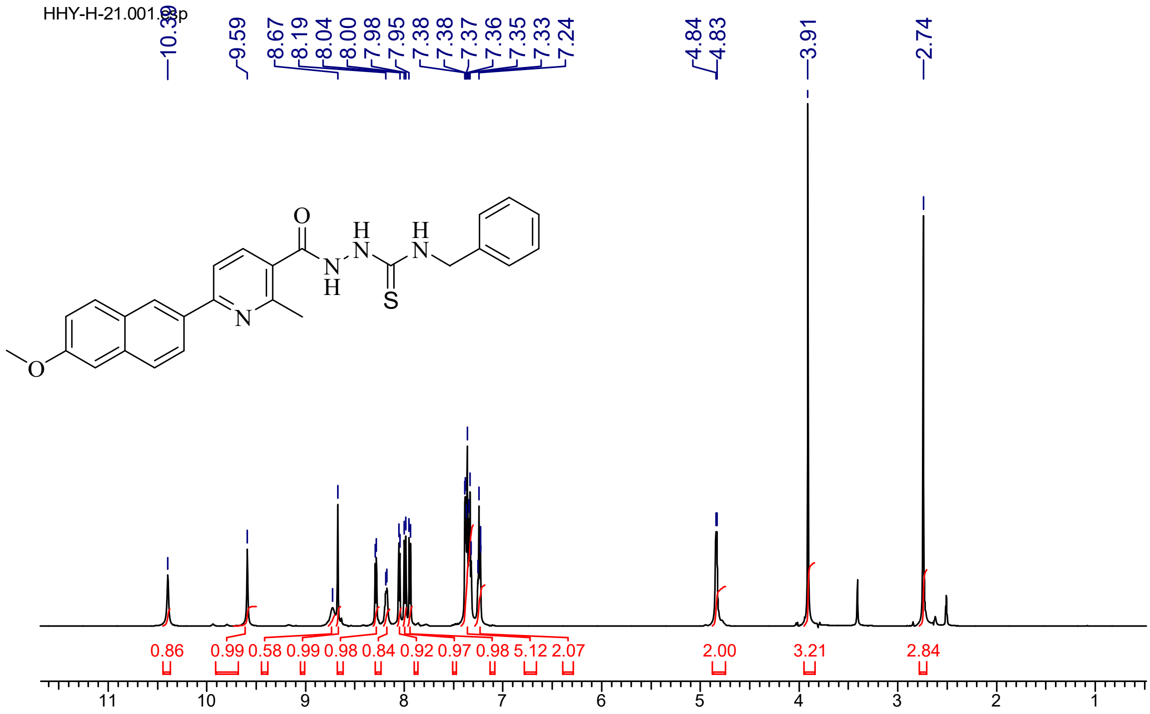

Figure S40. ^{1}H-NMR Spectrum of compound **9n.**

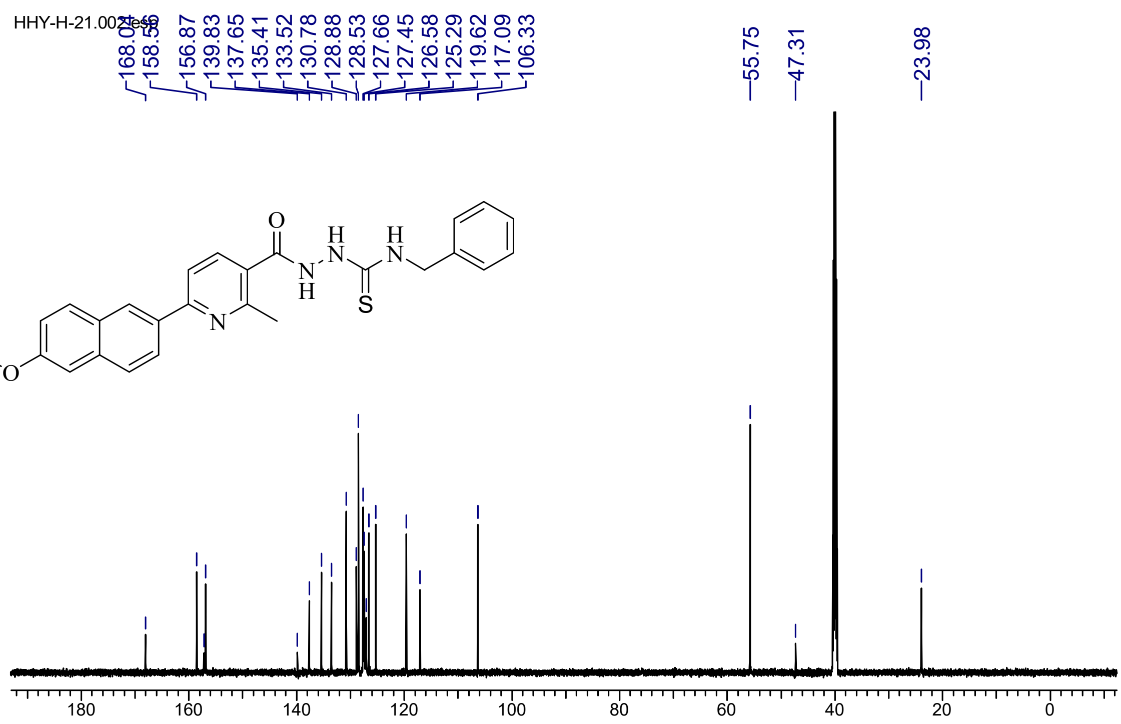

Figure S41. ¹C-NMR Spectrum of compound **9n.**

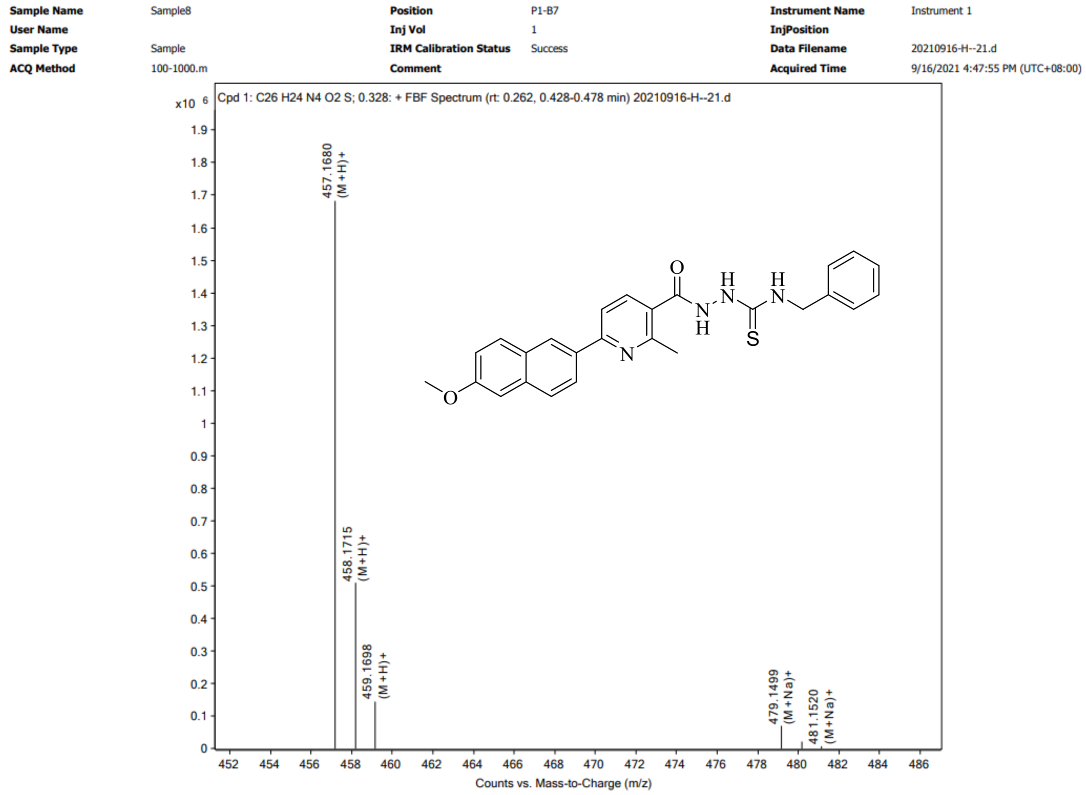

Figure S42. ESI-HRMS of compound **9n.**

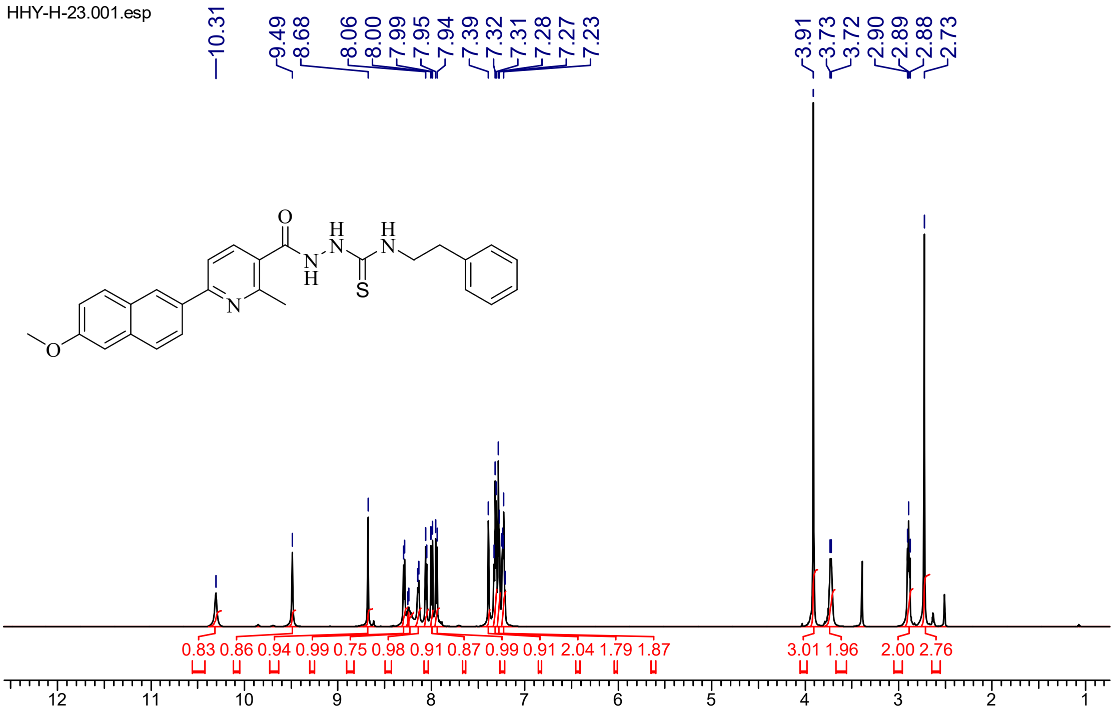

Figure S43. ^{1}H-NMR Spectrum of compound **9o.**

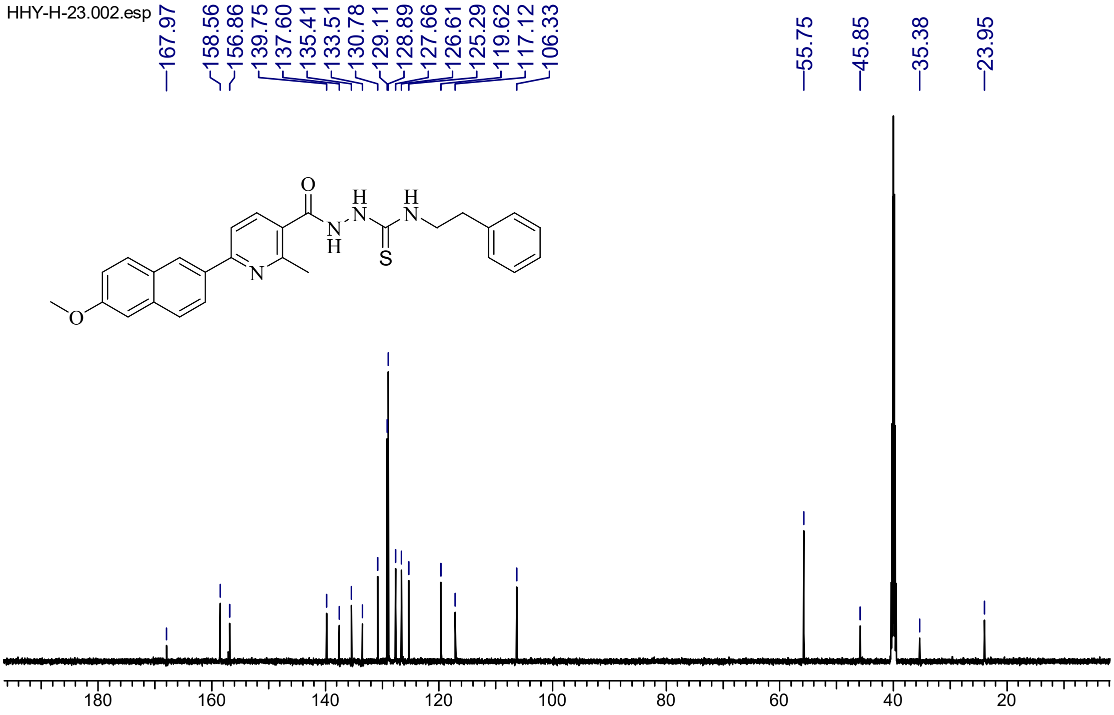

Figure S44. [1]C-NMR Spectrum of compound **9o.**

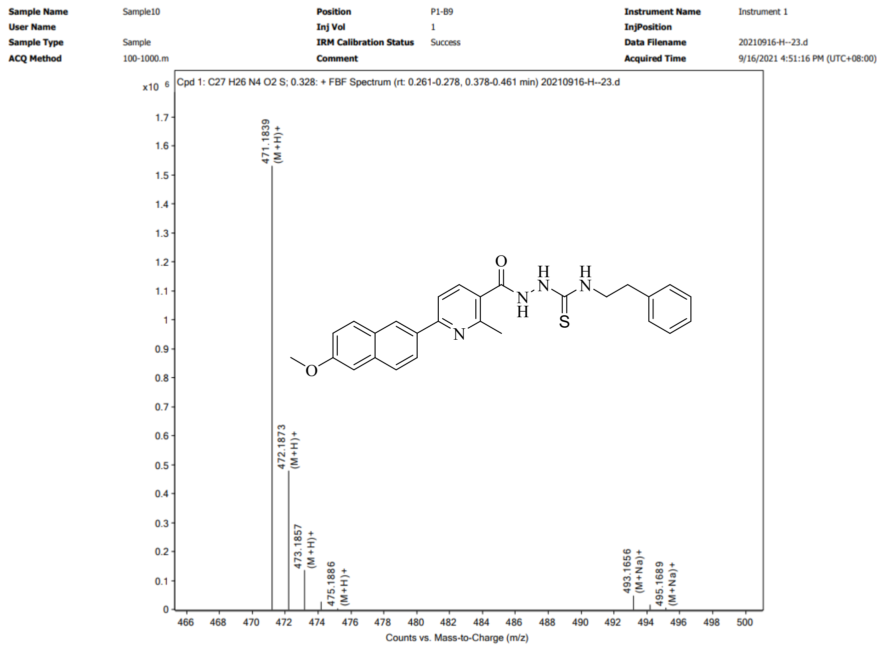

Figure S45. ESI-HRMS of compound **9o.**

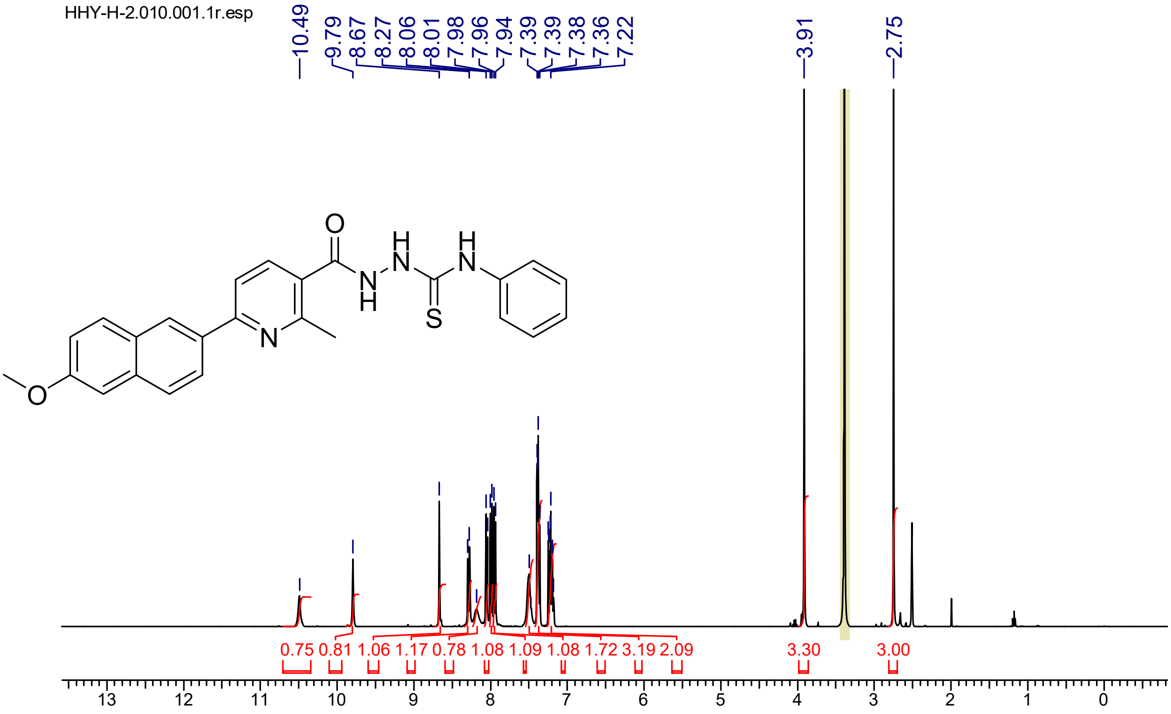

Figure S46. ^{1}H-NMR Spectrum of compound **9p.**

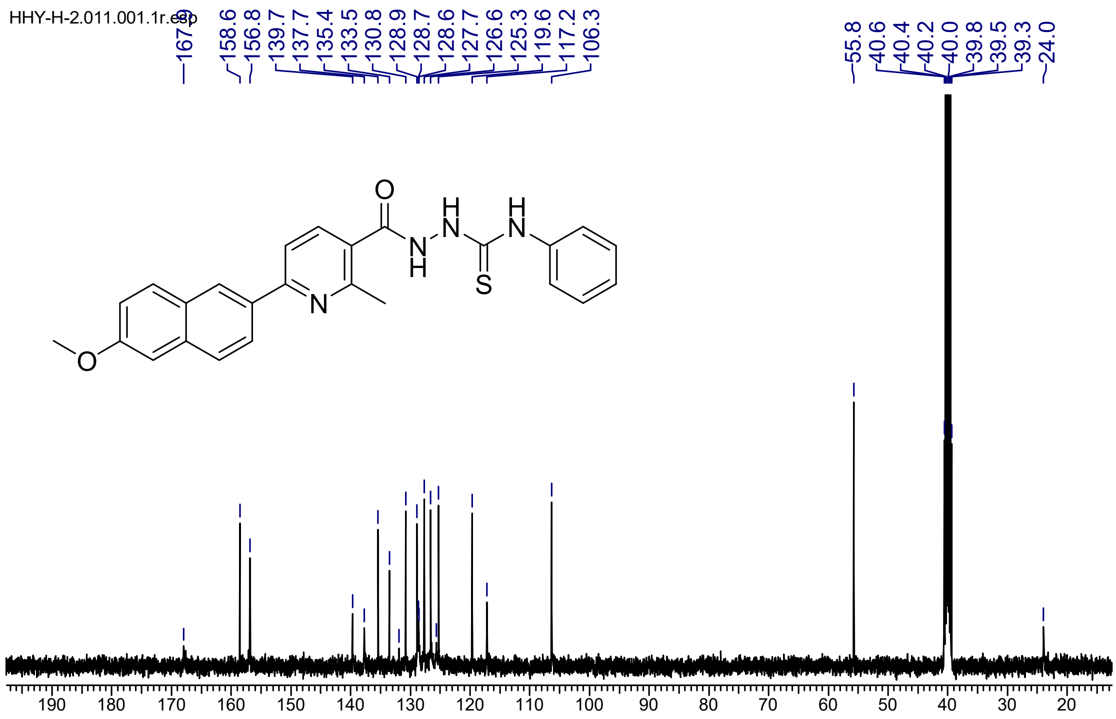

Figure S47. ^{1}C-NMR Spectrum of compound **9p.**

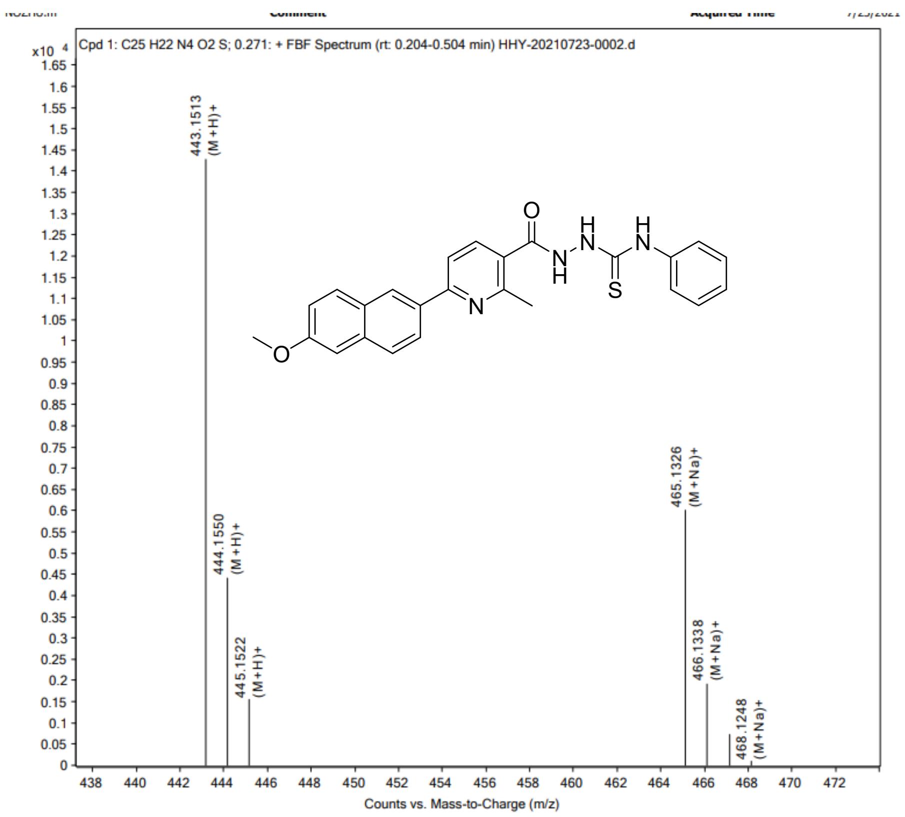

Figure S48. ESI-HRMS of compound **9p.**

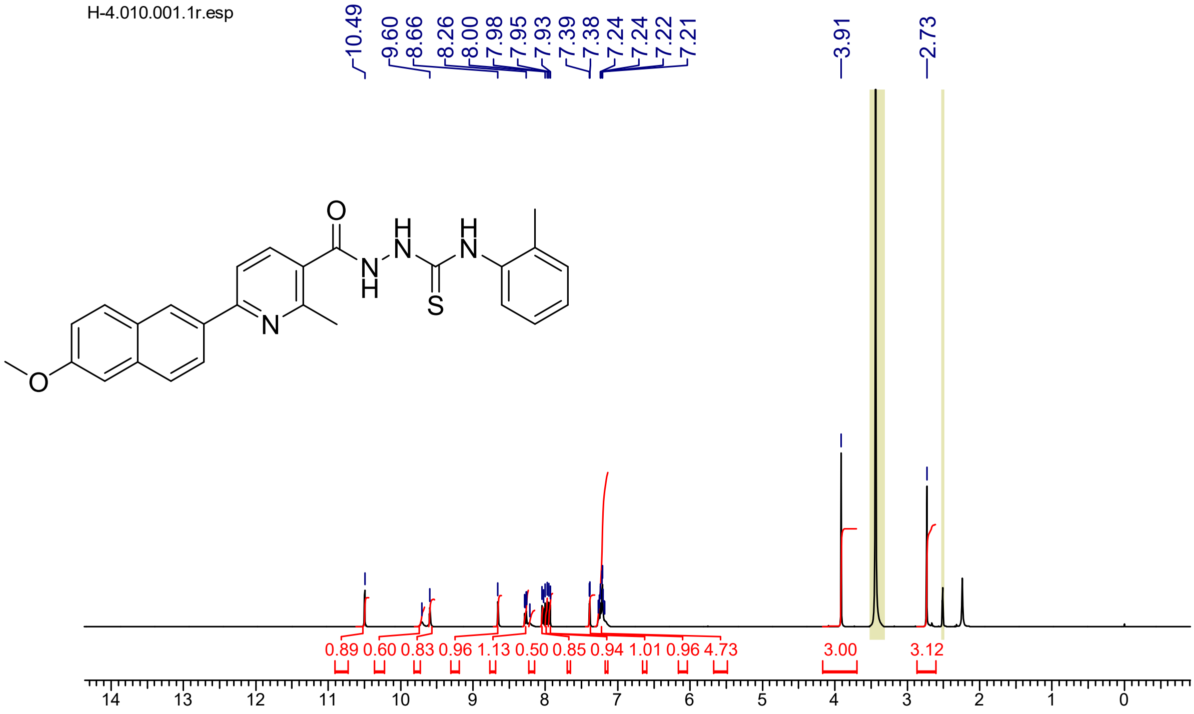

Figure S49. ^{1}H-NMR Spectrum of compound **9q.**

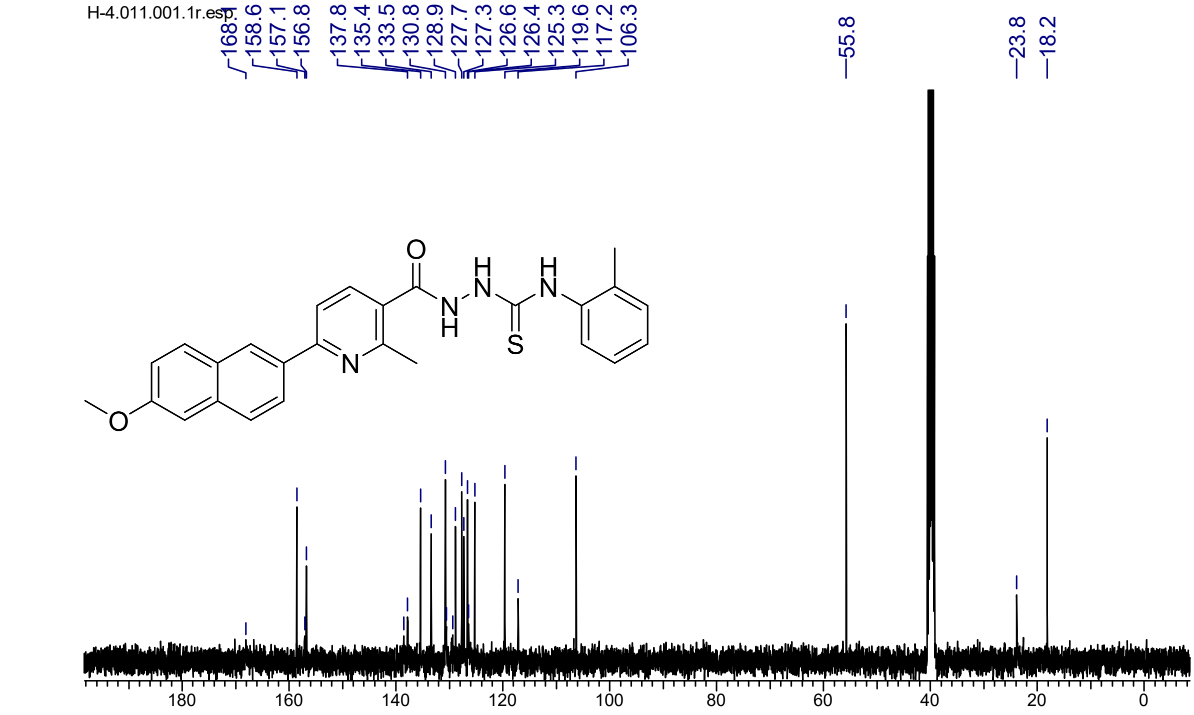

Figure S50. ^{1}C-NMR Spectrum of compound **9q.**

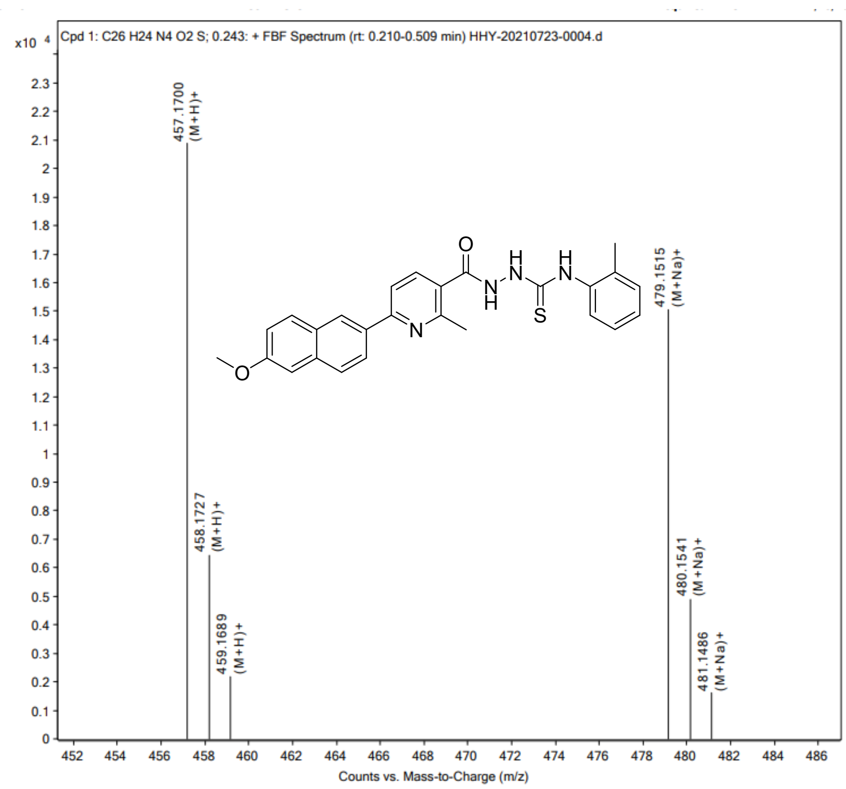

Figure S51. ESI-HRMS of compound **9q.**

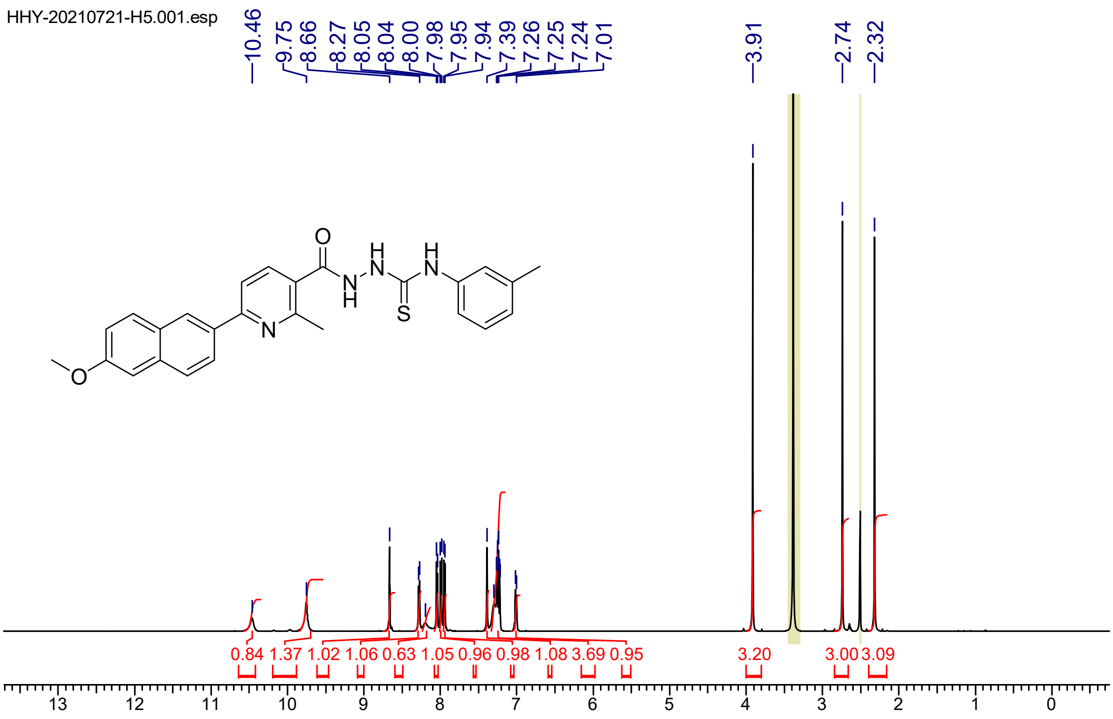

Figure S52. ¹H-NMR Spectrum of compound **9r.**

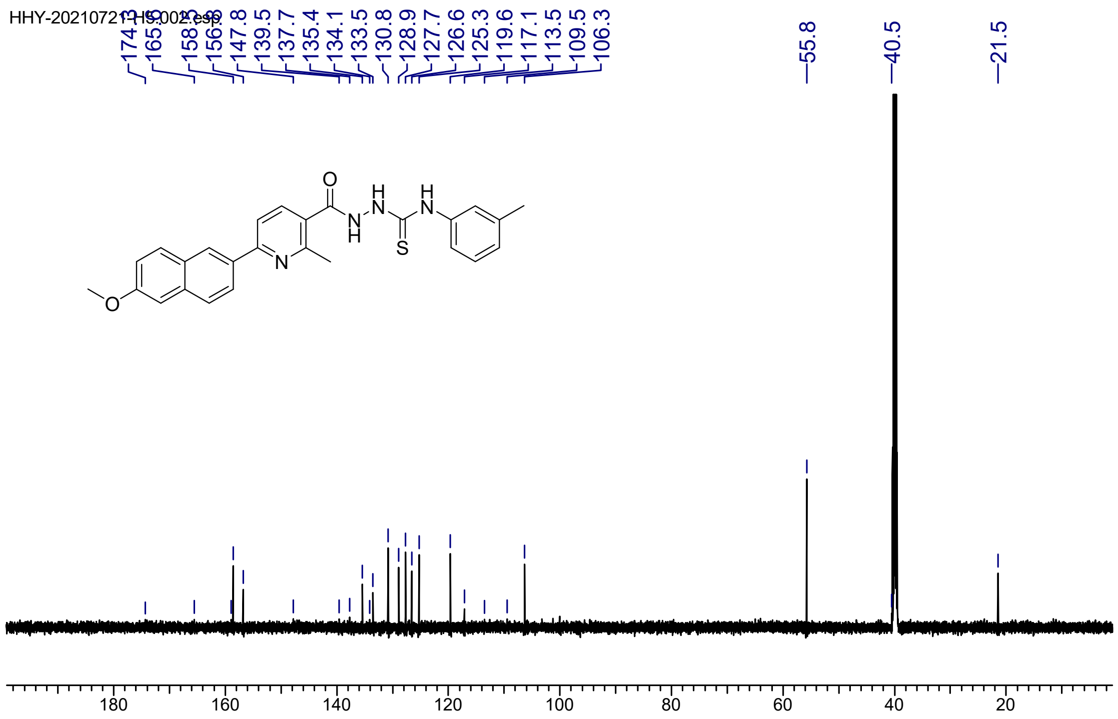

Figure S53. ^{1}C-NMR Spectrum of compound **9r.**

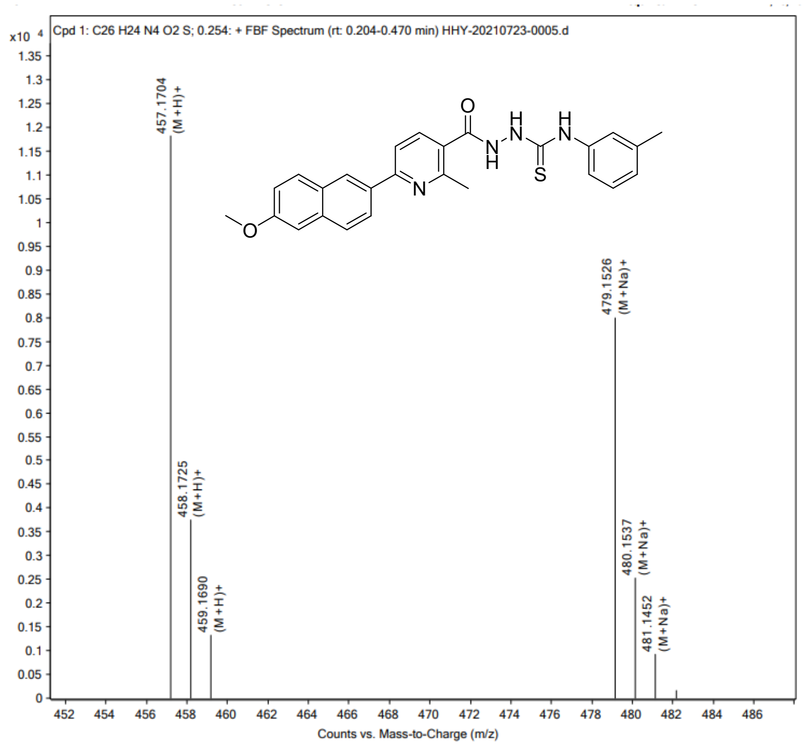

Figure S54. ESI-HRMS of compound **9r.**

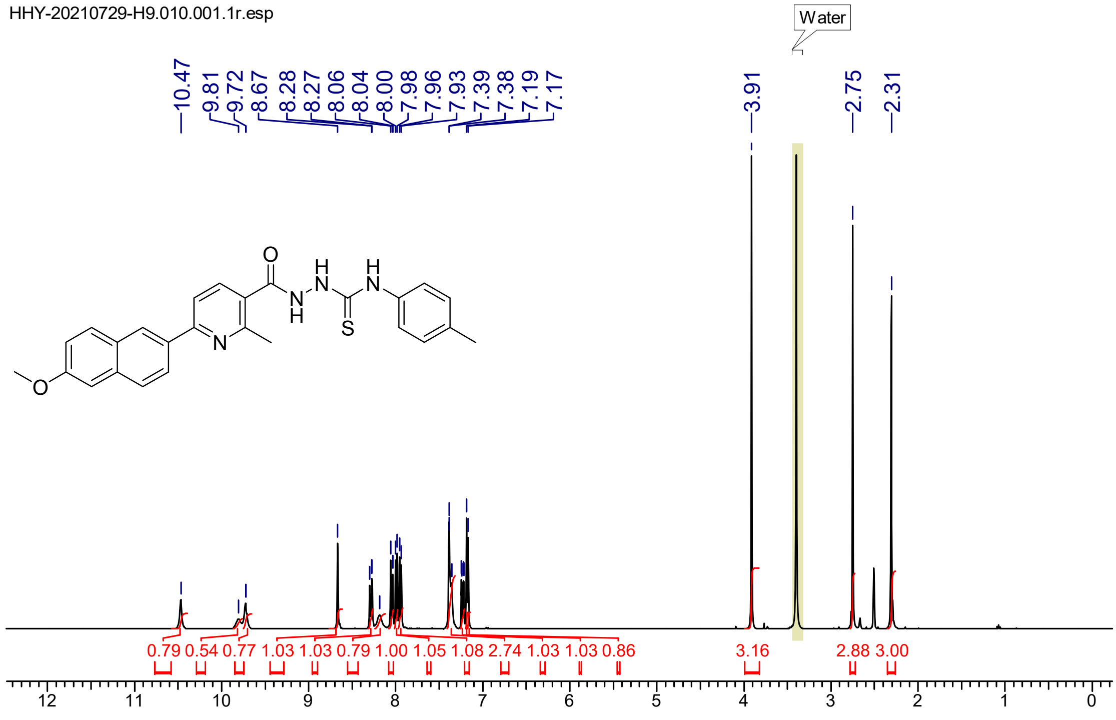

Figure S55. ^{1}H-NMR Spectrum of compound **9s.**

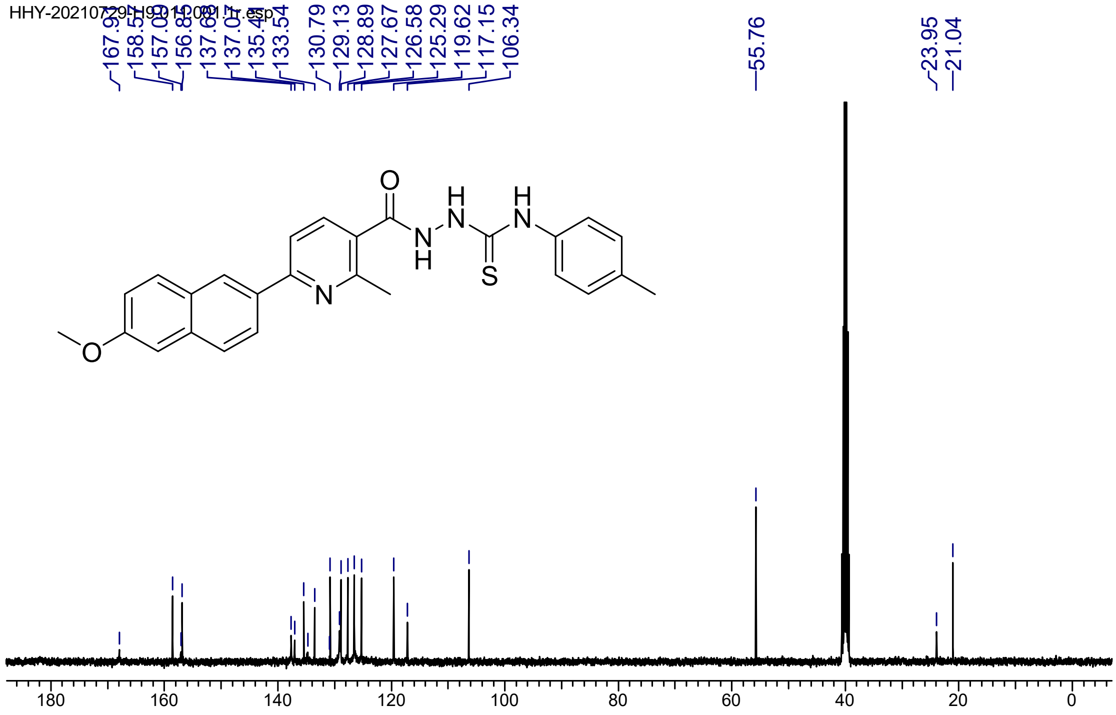

Figure S56. ^{1}C-NMR Spectrum of compound **9s.**

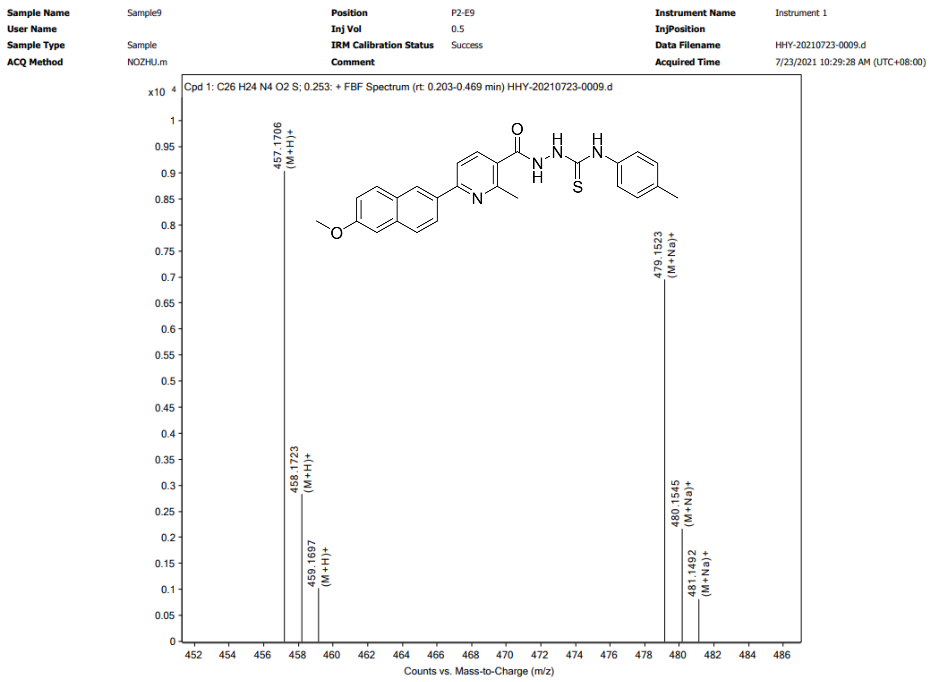

Figure S57. ESI-HRMS of compound **9s.**

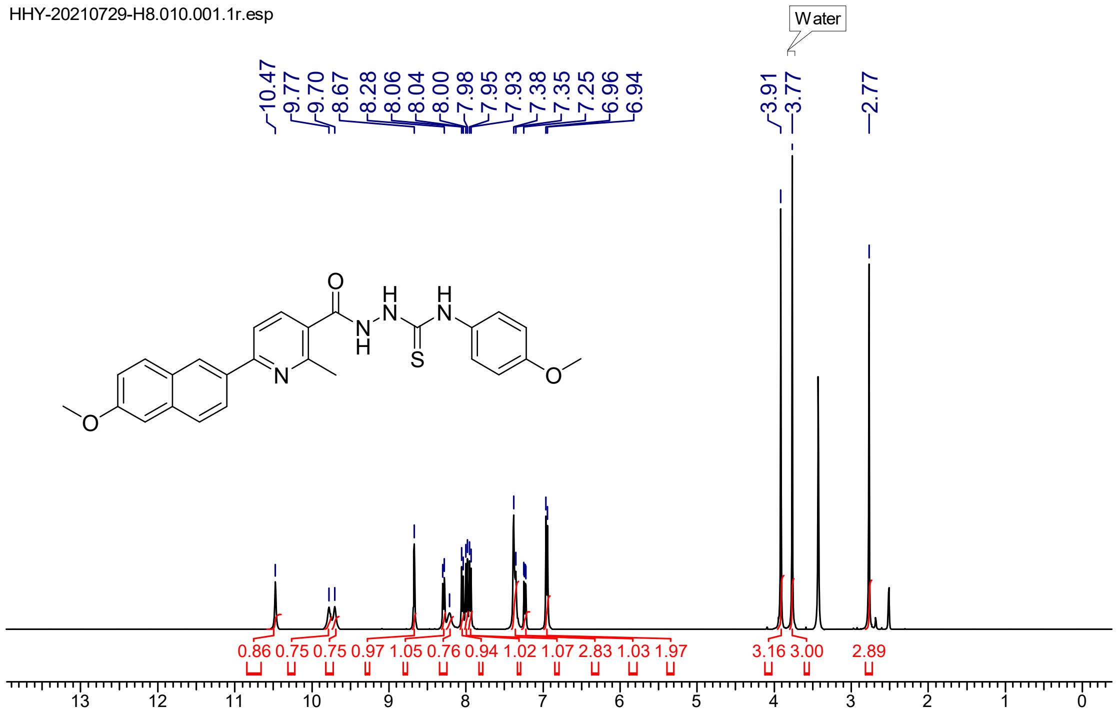

Figure S58. ^{1}H-NMR Spectrum of compound **9t.**

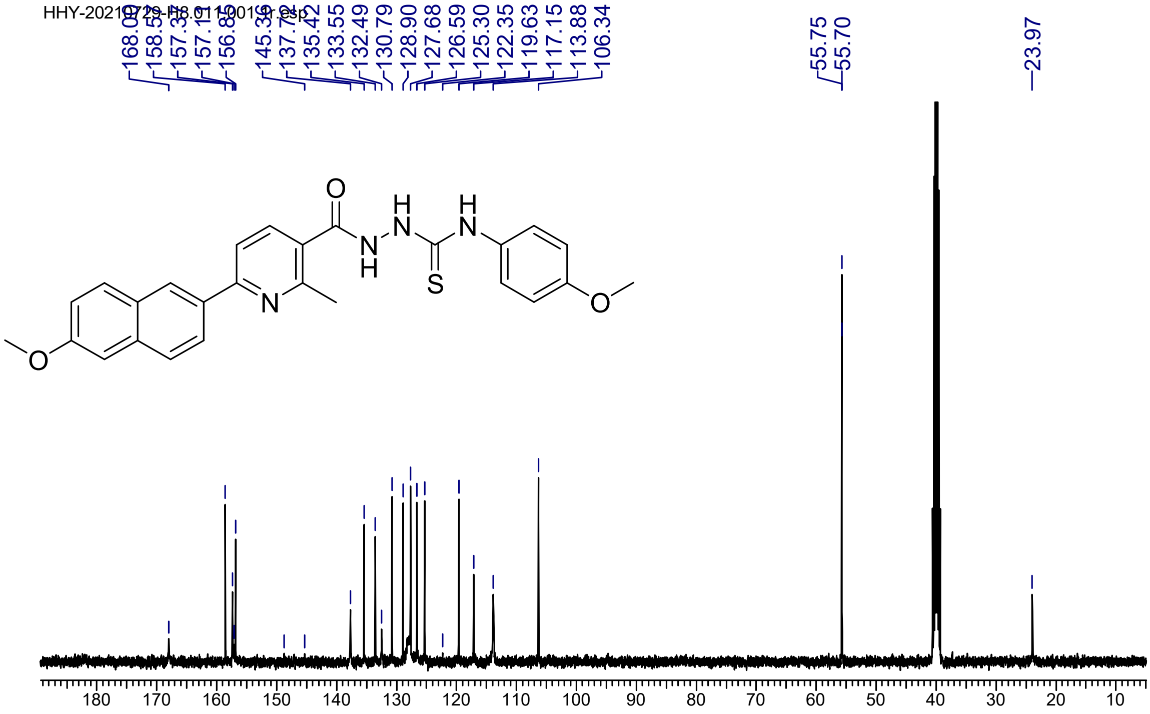

Figure S59. [1]C-NMR Spectrum of compound **9t.**

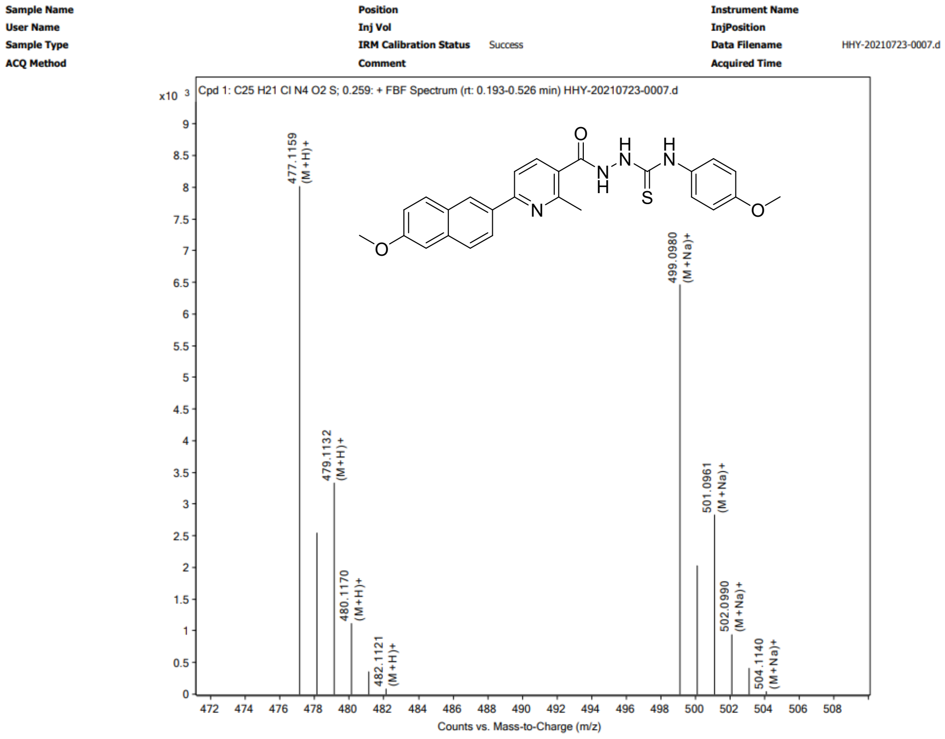

Figure S60. ESI-HRMS of compound **9t.**

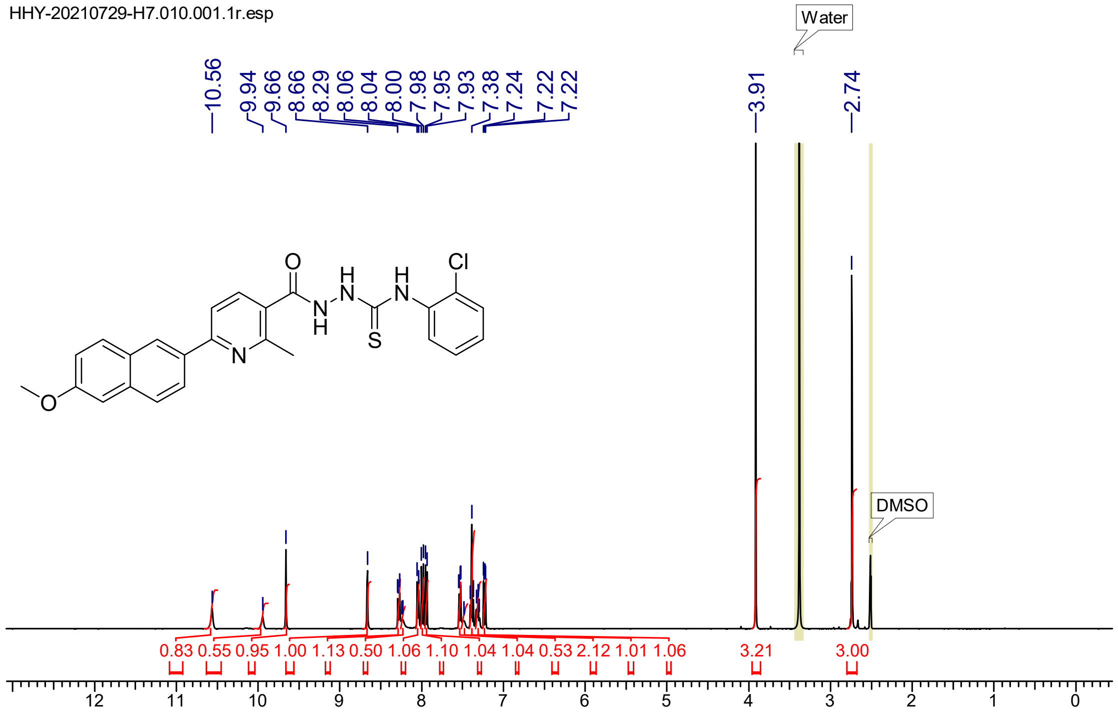

Figure S61. ¹H-NMR Spectrum of compound **9u.**

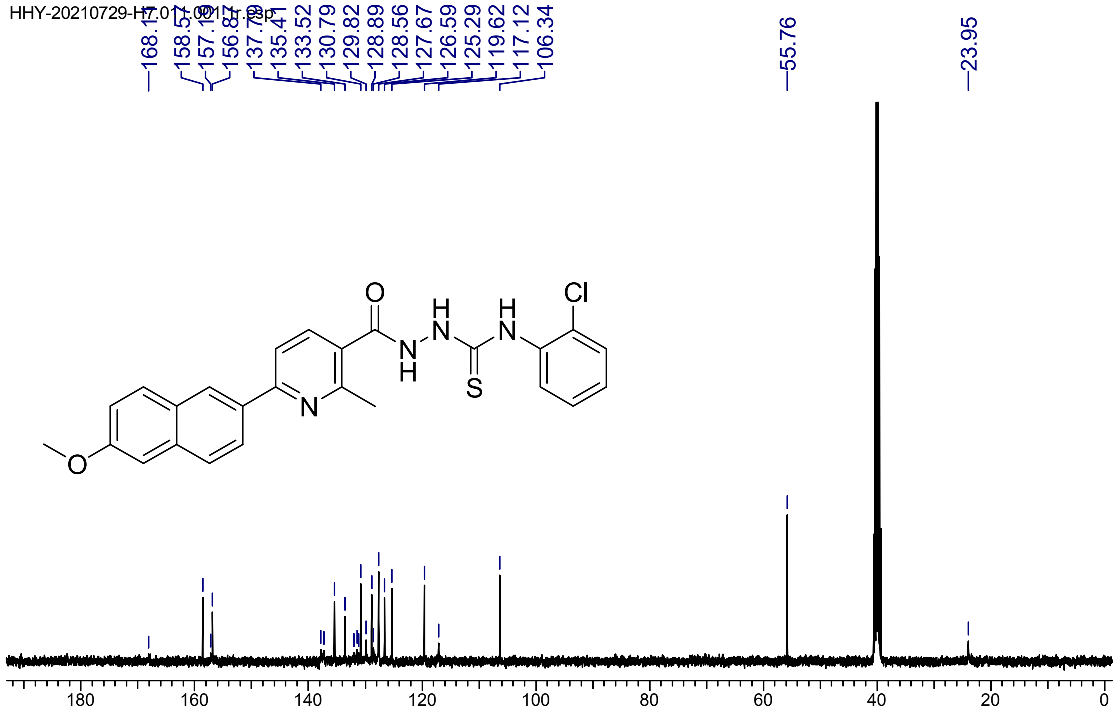

Figure S62. ¹C-NMR Spectrum of compound **9u.**

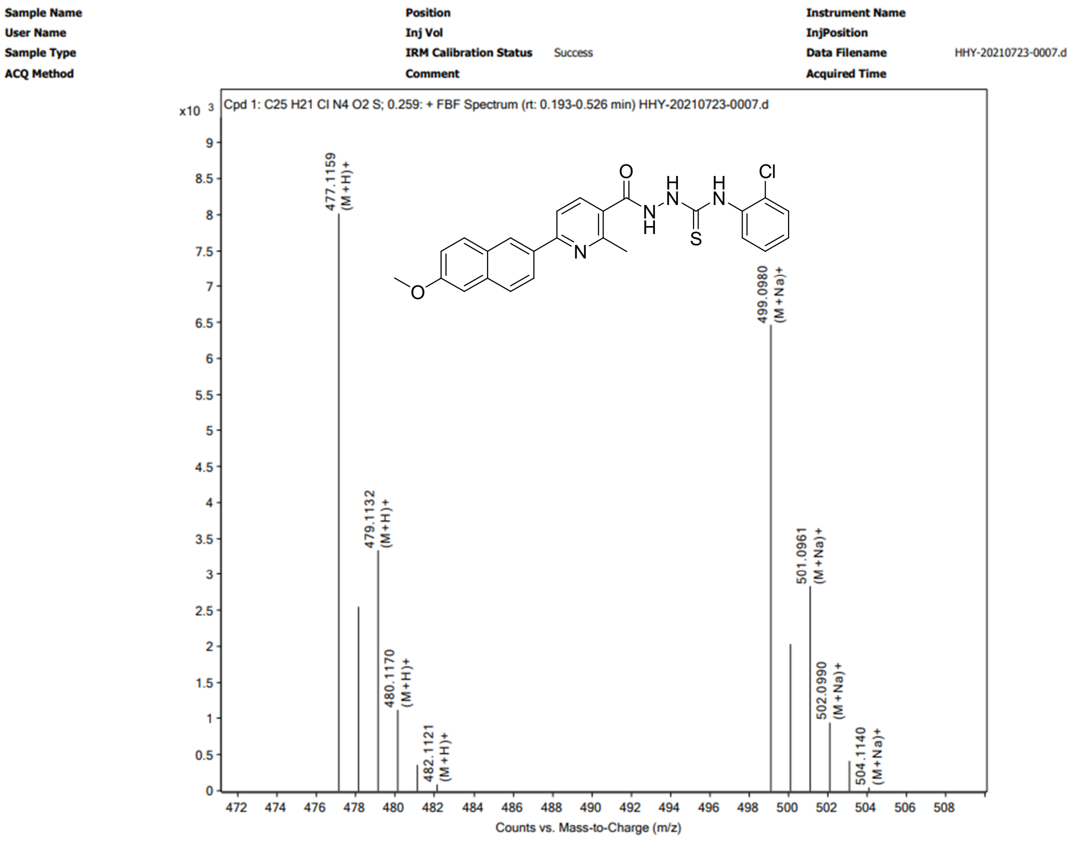

Figure S63. ESI-HRMS of compound **9u.**

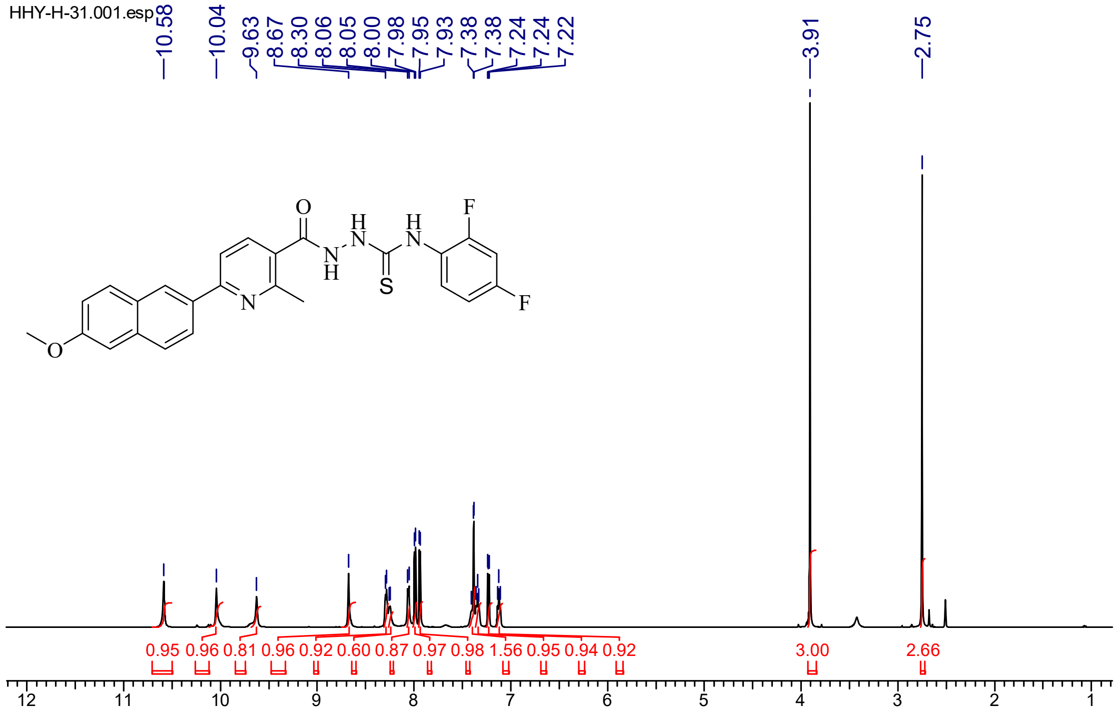

Figure S64. ^{1}H-NMR Spectrum of compound **9v.**

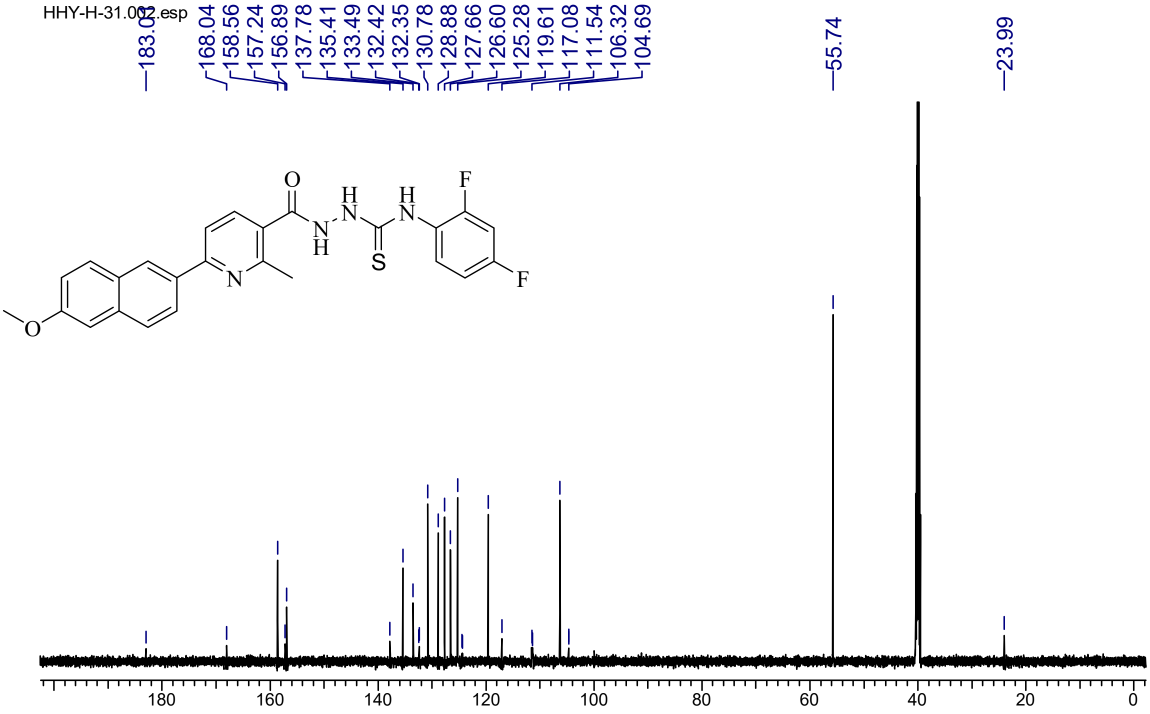

Figure S65. ^{1}C-NMR Spectrum of compound **9v.**

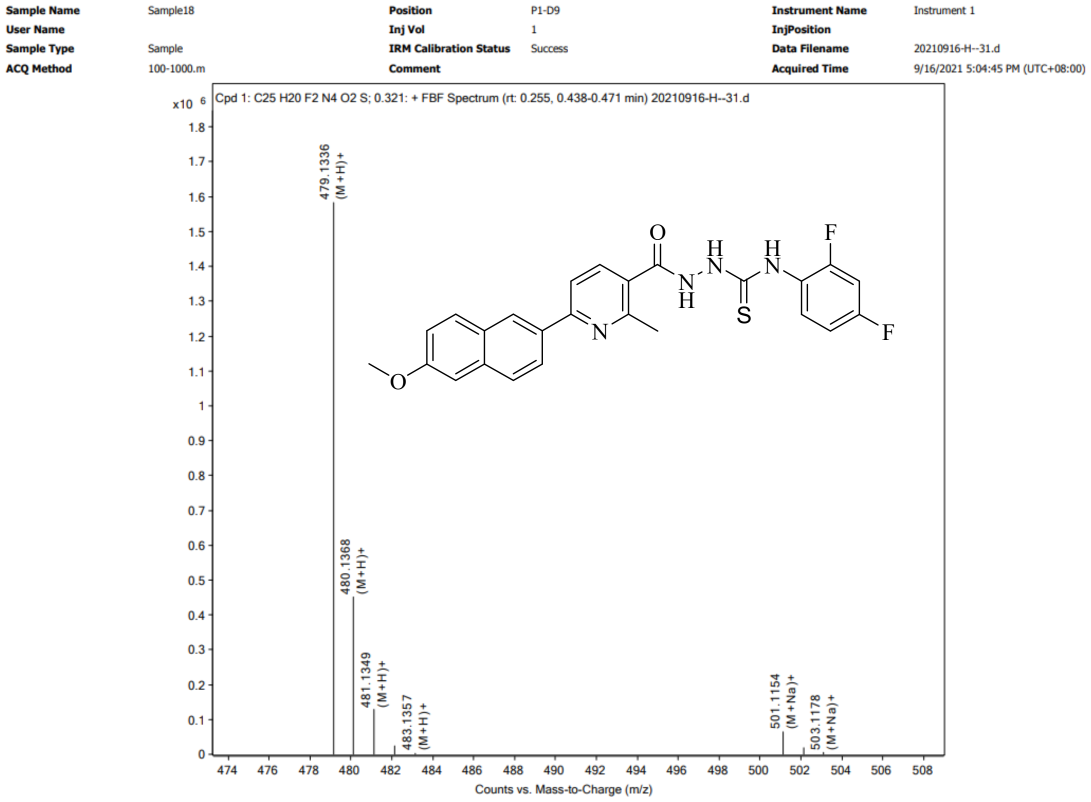

Figure S66. ESI-HRMS of compound **9v.**

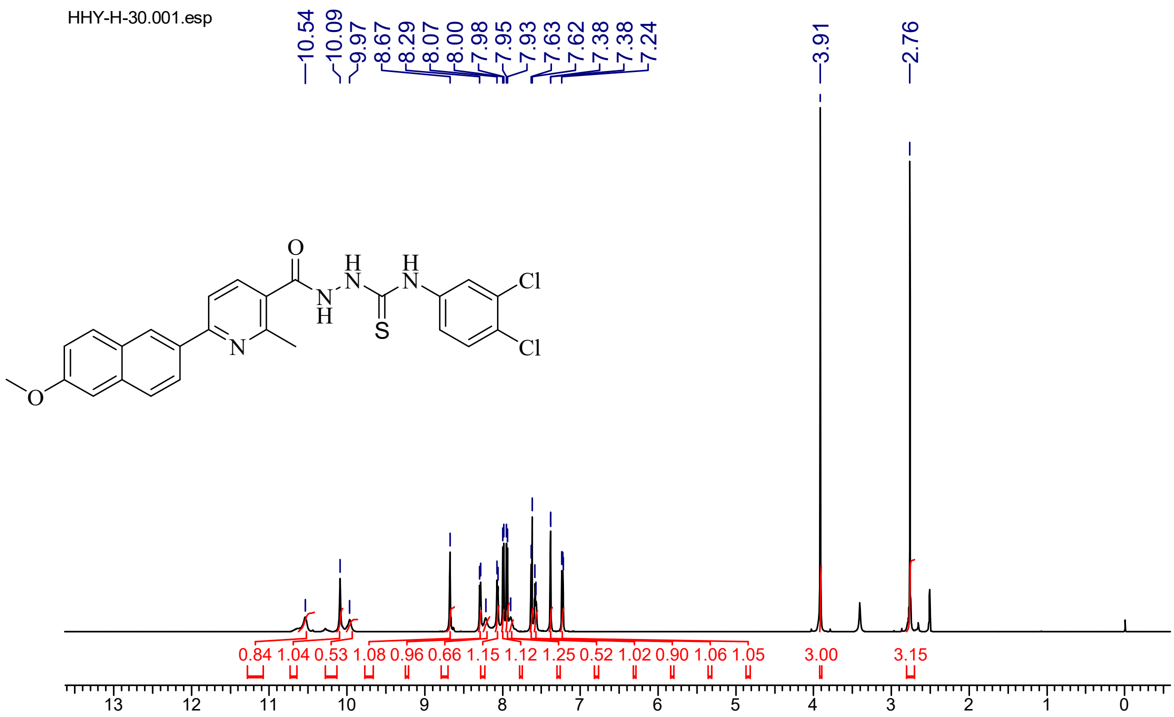

Figure S67. ^{1}H-NMR Spectrum of compound **9w.**

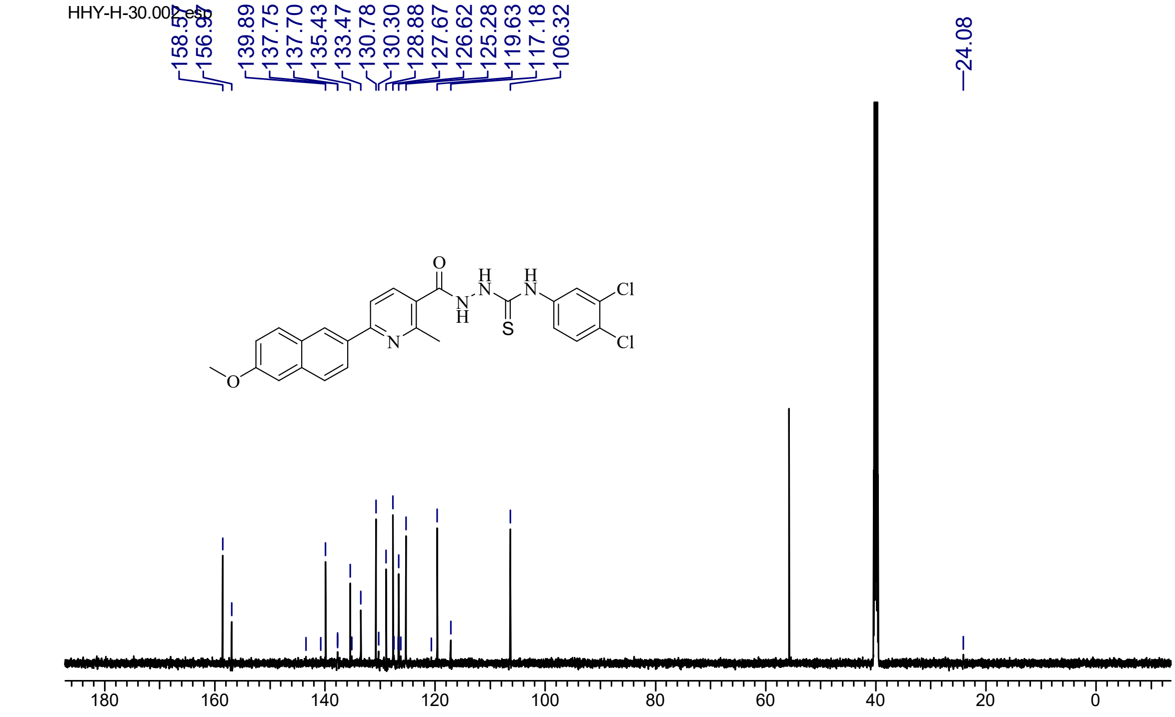

Figure S68. [1]C-NMR Spectrum of compound **9w.**

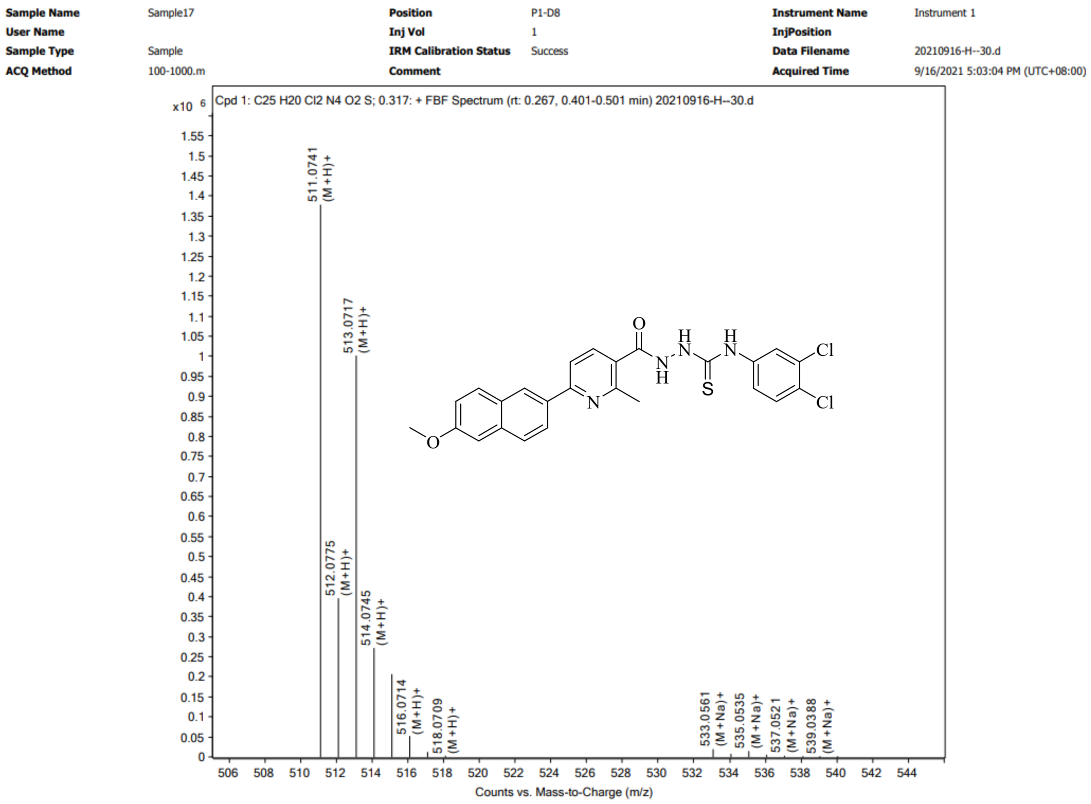

Figure S69. ESI-HRMS of compound **9w.**
